# Real-Time Binding Kinetics of Membrane Protein–Protein Interactions in a Membraneless Setting

**DOI:** 10.1021/acs.analchem.5c05510

**Published:** 2025-10-27

**Authors:** Yazheng Wang, Yalun Wu, Lauren A. Mayse, Danny Capucilli, Po-Jung J. Huang, Sekar Ramachandran, Soching Luikham, Jeung-Hoi Ha, Stewart N. Loh, Aaron J. Wolfe, Liviu Movileanu

**Affiliations:** † Department of Physics, 2029 Syracuse University, 201 Physics Building, Syracuse, New York 13244, United States; ‡ Department of Biomedical and Chemical Engineering, 2029 Syracuse University, 329 Link Hall, Syracuse, New York 13244, United States; § 558832 Ichor Life Sciences, Inc., 831 James Street, Syracuse, New York 13203, United States; ∥ The BioInspired Institute, 2029 Syracuse University, Syracuse, New York 13244, United States; ⊥ Department of Biochemistry and Molecular Biology, 12302 State University of New York−Upstate Medical University, 4249 Weiskotten Hall, 766 Irving Avenue, Syracuse, New York 13210, United States; # Department of Chemistry, State University of New York, College of Environmental Science and Forestry, 1 Forestry Dr., Syracuse, New York 13210, United States; △ Department of Biology, 2029 Syracuse University, 114 Life Sciences Complex, Syracuse, New York 13244, United States

## Abstract

A ubiquitous problem in protein analytics and medical biotechnology is assessing the interaction of a membrane protein receptor with its cognate protein ligand. This task generally requires transferring the receptor from native membranes or other expression host systems into supported lipid bilayers, liposomes, or nanodiscs. Such a reintegration process necessitates multiple steps for protein solubilization, renaturing, and functional reconstitution. Here, we opportunistically show that biolayer interferometry (BLI) can be directly utilized to evaluate the pre-equilibrium binding kinetics of a membrane protein receptor with its protein ligand in a label-free and membraneless setting. We present real-time measurements probing the association and dissociation phases of these transient complexes, conducted at a high signal-to-noise ratio using free proteomicelles in solution. As a proof-of-concept, we employ a subset of synthetic membrane proteins equipped with a programmable antibody mimetic binder that targets a specific protein ligand. Proteomicelles containing these binder-equipped membrane proteins exhibit high-affinity interactions with ligands attached to the sensor surface. These determinations are further validated by closely related surface plasmon resonance (SPR) measurements of the binder–ligand and proteomicelle–ligand interactions. Finally, this approach is amenable to high-throughput data collection, and its conceptual formulation is potentially extendable to other membrane proteins.

## Graphical abstract

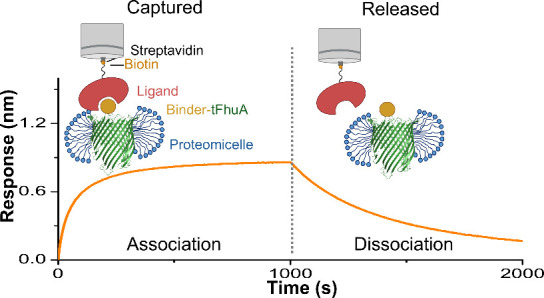

## Introduction

Membrane proteins play a crucial role in essential processes, including the transport of ions and metabolites, receptor–ligand interactions, enzymatic reactions, cell signaling, and intercellular communication.^1^ They represent a pivotal therapeutic target due to the opportunities to modulate their interactions with ligands using small-molecule drugs. Therefore, a mechanistic understanding of the binding kinetics of ligands with membrane proteins is critical for assessing cellular functions. Kinetic methods of protein interactions have traditionally employed radiolabeled and fluorescently labeled ligands.^2^ Real-time kinetic analysis employs gold-standard approaches, such as surface plasmon resonance (SPR)^3–5^ and biolayer interferometry (BLI).^6–8^ SPR and BLI are real-time, label-free, and high-throughput methods commonly used to determine the membrane receptor–ligand kinetics. In both cases, purified membrane proteins are functionally reconstituted into synthetic membrane systems, such as planar lipid bilayers,^5,9^ liposomes,^10,11^ or lipid nanodiscs,^12–14^ which are further immobilized onto the sensor surface. However, logistical challenges exist in measuring these interactions in lipids, such as a higher cost and a greater preparation time required to maintain a fully functional protein structure. There is a wide variety of affordable detergents carefully characterized for specific protein properties and available for standardized membrane protein reconstitution. Lipoparticles, such as lipid nanodiscs and liposomes, require an optimized detergent-to-lipid ratio for testing, followed by additional purification steps to eliminate empty lipid structures.^13^

Different groups have integrated creative technologies to address various obstacles in performing these kinetic determinations. For example, Ma and co-workers (2018) developed a technique in which human G-protein-coupled receptors (GPCRs) were displayed on viral envelopes of human herpes simplex virus-1 (HSV-1).^15^ These HSV-1 virions were immobilized on a gold-coated glass surface through flexible spacers. By applying an alternating electric field, the whole system was operated as an oscillator, whose amplitude changes served as an indicator of the binding kinetics of GPCRs with their ligands. In a different example, the SPR technology was recently expanded by depositing a silica surface onto the sensor chips.^16^ This approach led to the development of SPR imaging (SPRi), a surface-sensitive waveguide imaging method with enhanced measurement precision and compatibility with complex biological samples. In other instances, the developed assays can only determine affinity constants^17,18^ and do not facilitate the evaluation of the dissociation times of analytes and the efficacy of drug-driven inhibitions of the receptor activity.

In this article, we employ a membrane-free BLI approach without utilizing protein-containing lipoparticles to decrease experimental cost and optimization time. Such an experimental strategy would avoid any steps that may lead to the membrane receptor’s inactivation during its transfer and functional integration with the sensor surface. Here, the cognate ligand was chemically attached to the sensor surface (Figure 1a). Hence, detergent-solubilized membrane proteins freely interact in solution with the immobilized ligands, altering the sensor response. Here, we employed a subset of synthetic membrane proteins as the test case to show the proof-of-concept of this label-free approach. These synthetic membrane proteins comprise a bacterial outer membrane protein stem fused at its N-terminus to a protein binder via a flexible hexapeptide spacer. In this case, the membrane protein stem is the monomeric β barrel of ferric hydroxamate uptake component A of *Escherichia coli* (FhuA),^19,20^ an extensive truncation of the native protein (tFhuA) (Figure 1a).^21,22^

**Figure 1:**
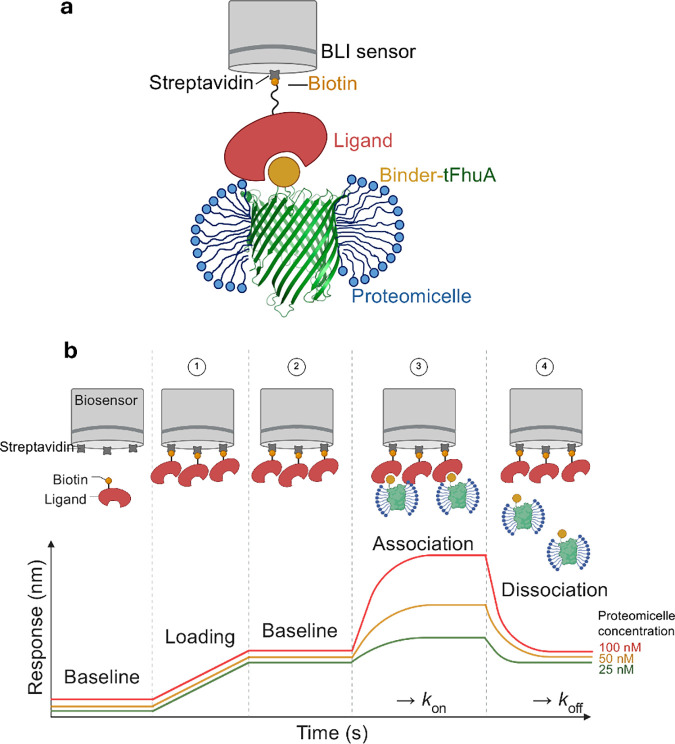
A method for determining the real-time binding kinetics between a detergent-refolded membrane protein and a protein ligand using biolayer interferometry (BLI). (a) An immobilized water-soluble protein ligand (dark pink) is loaded onto the BLI sensor (gray), while proteomicelles are in the well. These proteomicelles consist of a synthetic membrane protein equipped with a water-soluble antibody mimetic protein binder (yellow) fused to the N-terminus of the hydrophobic tFhuA outer membrane protein (green). The binder-tFhuA fusion protein is solubilized in detergent (blue). (b) Streptavidin-coated BLI biosensors were first loaded with biotin-labeled protein ligand for immobilization on the sensor surface. After the ligand loading, the BLI sensors were rinsed with the running buffer to remove any unbound ligand. For the association phase, the BLI sensors were dipped into 2-fold serial dilutions of the detergent-solubilized membrane protein (e.g., proteomicelles). The sensors were then transferred to a proteomicelle-free buffer to examine the dissociation phase. Loading-free BLI sensors were also run in parallel as controls.

The binder is a generalized antibody-mimetic protein scaffold,^23,24^ so these measurements were tested on various related cognate protein ligands, but with distinct interaction interfaces. The primary benefit of this design is its formulation as a single-polypeptide chain protein with a replaceable binder. Real-time binding kinetics between immobilized protein ligands and detergent-solubilized membrane proteins were monitored by measuring changes in the optical interference pattern of light reflected at the sensor surface (Figure 1b). Advantages of utilizing this method include clog-free sensor surfaces, the ability to handle crude samples, the analysis of challenging heterogeneous solutions, and a scalable setting for large-sample size screenings.

## Experimental Section

### The Design of Genes and Expression Constructs

The *zher2tfhua*, *zher3tfhua*, *egftfhua*, and *tgfαtfhua* pET-28a vector-containing plasmids were purchased from GenScript (Piscataway, NJ). These plasmids include 5′ to 3′-orientation genes encoding an affibody, *egf* or *tgfα*, a (GGS)_2_ flexible tether, and an extensive truncation of ferric hydroxamate uptake component A (*tfhua*) from *E. coli.* ^21,22^ The *zegfrtfhua* gene was generated using the *zher3tfhua* gene and employing site-directed mutagenesis with the Q5 mutagenesis kit (New England Biolabs, Ipswich, MA). Subsequently, the affibody genes *zegfr, zher2*, and *zher3* were produced using *zegfrtfhua, zher2tfhua*, and *zher3tfhua,* respectively, through the same site-directed mutagenesis approach with the addition of a six-histidine tag. Sequences of the forward and reverse primers utilized for these genetic modifications are provided in Table S1. The gene encoding Adnectin1-tFhuA, *adnectin1tfhua*, was generated in earlier studies.^25^ All plasmid sequences were checked through whole-plasmid DNA sequencing by MCLab (San Francisco, CA). All amino acid sequences of the proteins used in this study are provided in Table S2.

### Protein Expression and Purification

For the expression of binder-containing membrane proteins Z_EGFR_-tFhuA, Z_HER2_-tFhuA, Z_HER3_-tFhuA, Adnectin1-tFhuA, EGF-tFhuA, and TGFα-tFhuA, the plasmids were transformed into *E. coli* BL21­(DE3) cells (New England Biolabs) and grown in 1 L Luria– Bertani (LB) medium at 37 °C until the OD_600_ reached a value of ∼0.5. Here, TGFα also includes the remaining domain of the extracellular region of its precursor.^26^ Then, cells were induced using 1 mM isopropyl β-d-1-thiogalactopyranoside (IPTG). Cells were cultured for ∼4 h at 37 °C, then harvested by centrifuging at 3500*g* for 30 min at 4 °C. These steps were followed by resuspension in the lysis buffer containing 300 mM KCl, 50 mM Tris-HCl, 5 mM ethylenediaminetetraacetic acid (EDTA), pH 7.5. Cells were lysed using a microfluidizer (Model 110L; Microfluidics, Newton, MA), then centrifuged at 108 000*g* for 30 min at 4 °C. The supernatant was discarded, and the pellet was resuspended in 1% (v/v) Triton X-100 buffer containing 1 mM EDTA, homogenized, then centrifuged at 108 000*g* for 30 min at 4 °C. This process was repeated three times. The insoluble pellet was dissolved in 8 M urea. Further protein purification was conducted by fast protein liquid chromatography (FPLC) using a next-generation chromatography (NGC) Quest 10 Plus System (Bio-Rad, Hercules, CA) equipped with an ion-exchange column (5 mL, Foresight Nuvia Q Column; Bio-Rad). The protein was eluted using a 10-column volume linear gradient of KCl, ranging from 0 to 1 M, in a buffer containing 20 mM Tris-HCl, 8 M urea, pH 7.5. The protein purity and size were confirmed using an SDS-PAGE gel analysis.

The affibody binders Z_EGFR_, Z_HER2_, and Z_HER3_, as well as monobody Adnectin1, were expressed using *E. coli* BL21­(DE3). All transformed cells were grown in LB medium at 37 °C until the OD_600_ reached 0.5, then induced with 0.1 mM IPTG and grown for 4 h at 37 °C. The cells were harvested by centrifugation at 3500*g* for 30 min at 4 °C. The cell pellet was resuspended in lysis buffer containing 300 mM KCl, 20 mM Tris, pH 7.5, 3 mM dithiothreitol (DTT), 20 mM imidazole, 0.1 mM phenylmethylsulfonyl fluoride (PMSF), and a cocktail of EDTA-free protease inhibitor (cOmplete, Sigma-Aldrich, St. Louis, MO). We utilized a microfluidizer (Model 110L, Microfluidics) to lyse the cells, then centrifuged at 108 000*g* at 4 °C for 30 min. Then, the supernatant was collected and filtered using a 0.22-μm filter. Further purification by FPLC system using an immobilized metal-affinity column (5 mL, EconoFit Profinity IMAC; Bio-Rad, Hercules, CA). The protein was eluted using a 10-column linear imidazole gradient in a buffer of 300 mM KCl, 20 mM Tris-HCl, 3 mM DTT, 500 mM imidazole, pH 7.5. The peak fractions containing the target protein were verified for purity by performing an SDS-PAGE gel analysis.

The ectodomains of the epidermal growth factor receptor (EGFR), human epidermal growth factor receptor 2 (HER2), and human epidermal growth factor receptor 3 (HER3) were expressed in Expi293F cells (Thermo Fisher Scientific; Catalog No. A14527) using polyethylenimine (PEI)-mediated transfection. The cells were cultured in 1 L batches for 5 days in Dynamis Growth Medium (Gibco, Thermo Fisher Scientific, Pittsburgh, PA), during which the target proteins were secreted into the culture medium. The supernatant was treated with 5 mL of 1 M Tris-HCl pH 8.0 and 125 μL of CaCl_2_, then loaded onto a 1 mL HIStrap HP immobilized metal-affinity column (GE Healthcare Life Sciences, Pittsburgh, PA). This column was pre-equilibrated with a buffer containing 50 mM Tris-HCl, 500 mM NaCl, 10% glycerol, 10 mM imidazole, and pH 8.0. The bound protein was eluted using a 100 mL linear gradient from 0% to 100% of an elution buffer containing 50 mM Tris-HCl, 500 mM NaCl, 10% glycerol, 500 mM imidazole, pH 8.0. The peak fractions containing the target protein were checked for purity by performing an SDS-PAGE gel analysis. Pooled fractions were dialyzed overnight in 2 L of 20 mM HEPES, 50 mM NaCl, 5% glycerol, pH 8.0. The protein sample was concentrated using a 10 kDa molecular weight cutoff concentrator and flash-frozen in 100 μL aliquots.

### Membrane Protein Refolding

The binder-containing tFhuA membrane proteins were diluted to a concentration of ∼25 μM in 8 M urea, and *n*-dodecyl-β-d-maltopyranoside (DDM) (Anatrace, Maumee, OH) was added to reach a final concentration of 1% (w/v).^27^ Then, the sample was dialyzed using a 14 kDa molecular weight cutoff bag in 4 L of the refolding buffer containing 200 mM KCl, 20 mM Tris-HCl, pH 7.5, at 4 °C for 24 h. Then, the dialysis process was repeated in 1 L fresh refolding buffer every 24 h four times. The dialysis process corresponded with the final urea concentrations of 4 mM, 8 μM, 12 nM, and 12 pM. The refolded protein sample was centrifuged at 3500*g* for 2 min at 4 °C to remove any remaining protein precipitates. The refolded protein was in the supernatant. The concentration of the protein sample was assessed using the molar absorptivity at a wavelength of 280 nm.

### Circular Dichroism for Confirming Native Membrane Protein Folding

Far-ultraviolet (UV) circular dichroism (CD) spectra curves were collected for each membrane protein and urea concentration. All CD experiments were conducted at 22 °C. A 1 mm cuvette and an Aviv model 420 CD spectrometer (Lakewood, NJ) were used. The absorption was scanned from 215 nm to 250 nm at 8 M urea, and at all other urea concentrations from 210 nm to 250 nm. The molar ellipticity, [θ], was determined by normalizing each millidegree of differential absorption by the molar concentration and number of amino acids of the protein sample.^28,29^ The CD readings of the melting curves were conducted at a wavelength of 219 nm, because these membrane proteins have a significant β-barrel structure.^30^ Temperature dependence experiments were performed at temperatures ranging from 20 °C to 90 °C in 2 °C increments.

### Biolayer Interferometry Determinations

Biolayer interferometry (BLI) experiments were run by the Octet Red384 platform (FortéBio, Fremont, CA).^31,32^ The protein ligands or protein binders were biotinylated at the N-terminus for immobilization onto streptavidin-coated BLI sensors. This process was conducted through the reaction of these proteins with sulfosuccinimidyl-6-[biotinamido]-6-hexanamido hexanoate (EZ Link Sulfo-NHS-LC-LC-Biotin, Thermo Scientific, Rockford, IL). To remove the unbound biotin from the biotinylation reaction of the binder or the ligand, a PD-10 desalting column (Cytiva, Marlborough, MA) or a Spin-X UF concentrator (Corning, Corning, NY) was employed, respectively. For experiments in which the protein ligands were immobilized onto the sensor surface, the presoaking and running buffers included 300 mM KCl, 20 mM Tris-HCl, 0.2% (w/v) DDM, 1 mg/mL bovine serum albumin (BSA), and pH 7.5. The presoaking was conducted for 15 min. For experiments without proteomicelles, the presoaking and running buffers were the same as above, but without DDM. During the association phase, sensors were immersed in wells containing either proteomicelles or the protein ligands, while in the dissociation phase, sensors were transferred to analyte-free wells. The response curves were baseline-corrected by subtracting signals obtained from reference wells, which contained protein ligand- or protein binder-free solutions that were run in parallel. The reported data represent the results of three independent BLI measurements. The binding curves were analyzed further and fitted using Octet Data Analysis software (FortéBio). The association curves were fitted using the following equation:^33,34^

$$Y=Y_{\infty }-\left(Y_{\infty }-Y_{0}\right)e^{-k_{\mathrm{obs}}t}$$ 1

Here, *Y*_0_ and *Y*_∞_ are the association phase response signal values at the initial and end of the reaction, *t* indicates the cumulative time of the association phase, and *k*_obs_ represents the apparent first-order reaction rate constant for the association phase. The curves of the dissociation phase were fitted using the following equation:

$$Y=Y_{\infty }+\left(Y_{0}-Y_{\infty }\right)e^{-k_{\mathrm{off}}t}$$ 2

Here, *Y*_0_ and *Y*_∞_ are the dissociation phase response signal values at the initial and end of the dissociation process, and *k*_off_ indicates the dissociation rate constant. Finally, the association rate constant (*k*_on_) was determined using the slope of the linear curve:^34,35^

$$k_{\mathrm{obs}}=k_{\mathrm{on}}\left[C\right]+k_{\mathrm{off}}$$ 3

The equilibrium dissociation constant (*K*_D_) was indirectly determined using the following equation:

$$K_{D}=\frac{k_{\mathrm{off}}}{k_{\mathrm{on}}}$$ 4

All BLI measurements were performed at 22 °C.

### Surface Plasmon Resonance Measurements

All surface plasmon resonance (SPR) experiments^31,36,37^ were conducted on a Cytiva Biacore 8K Plus (Cytiva Life Sciences, Marlborough, MA). All buffers and dilutions were prepared using ultrapure water obtained from an IQ 7000 Milli-Q system (Millipore Sigma, Burlington, MA). EGFR, HER2 and HER3 proteins were immobilized onto the active flow cell of each channel of a Cytiva Series S Sensor Chip CM5 (Cytiva Life Sciences). A CM5 chip was inserted into the instrument and equilibrated for 1 h at 25 °C in PBS-P+ running buffer, which consisted of 20 mM sodium phosphate, 137 mM NaCl, 2.7 mM KCl, and 0.05% Tween 20, at pH 7.5. The chip surface was activated with a 420-s injection of a 1:1 mixture of *N*-hydroxysuccinimide (NHS) and 1-ethyl-3-(3-(dimethylamino)­propyl)­carbodiimide (EDC) (Cytiva Amine Coupling Kit, Cytiva Life Sciences) at 10 μL/min across both active and reference flow cells. This activation process was followed by a wash of the microfluidics with 1 M ethanolamine-HCl, pH 8.0.

Following activation, EGFR, HER2, and HER3 (5 μg/mL; analyte-dependent) in 50 mM sodium phosphate, 50 mM NaCl, pH 4.0, were injected across the active flow cell for 270 s at 5 μL/min. Following ligand immobilization, both active and passive flow cells were chemically deactivated with a 420-s injection of 1 M ethanolamine-HCl, pH 8.0, at 10 μL/min. All analytes were prepared in the same manner as for the BLI assay. Z_EGFR_ and Adnectin1 were titrated from 400 nM, while Z_HER2_ and Z_HER3_ were titrated from 50 nM. Single-cycle kinetic analyses were conducted at a flow cell and sample compartment temperature of 25 °C in a running buffer composed of 20 mM Tris-HCl, 300 mM KCl, 1% (w/v) BSA, and 0.05% (v/v) Tween 20, pH 7.5. All analyte injections consisted of a 2-fold, nine-point dilution series with a 120-s association time, a 1200-s dissociation time, and a flow rate of 50 μL/min. Before curve fitting, all data generated from the active flow cell of each channel were double-referenced to both the appropriate buffer blanks and the reference flow cell. For all data, the equilibrium dissociation constant (*K*_D_) was calculated directly using the equation *K*_D_ = *k*_off_ /*k*_on_. All interactions were independently determined in triplicate. Experimental data and fits were plotted using GraphPad Prism 8 (GraphPad Software, Boston, MA).

### Molecular Graphics

All cartoons showing molecular graphics were prepared using PyMOL (Version 2.4.0; Schrödinger, LLC). Entries 3MZW.pdb, 3QWQ.pdb, 1NQL.pdb, 7SZ7.pdb, and 1BY3.pdb from Protein Data Bank were used for visualizations and molecular graphics of Z_HER2_–HER2_,_ ^38^ Adnectin1-EGFR,^39^ EGF-EGFR,^40^ TGFα-EGFR,^41^ and FhuA,^19^ respectively. Figure 1b was designed and created using BioRender (Toronto, Canada).

## Results and Discussion

### Development of Synthetic Membrane Proteins

In this study, we used protein engineering to develop synthetic membrane proteins with a programmable protein binder^42,43^ against targeted protein ligands. The membrane protein–protein binding kinetics were probed for various ligands while keeping the membrane protein stem unchanged. Our synthetic membrane proteins were expressed and purified as single-polypeptide chain proteins. The protein binder was an antibody mimetic scaffold in the form of an affibody,^44,45^ or a monobody.^25,46^ In this way, we created tFhuA-based membrane proteins against the ectodomains (e.g., ECD, the extracellular domains) of the human epidermal growth factor receptors EGFR/HER1, HER2, and HER3 (Tables S2–S5). An affibody is a 58-residue three-helix bundle protein based on the Z domain of staphylococcal protein A.^42,45^ The tFhuA stem is highly acidic, likely due to negative side-chain charges on the loops, β-barrel turns, and pore lumen.

We employed the Z_EGFR_,^47^ Z_HER2_,^38^ and Z_HER3_ ^48^ affibodies against EGFR/HER1, HER2, and HER3, respectively. Each affibody was fused to the N terminus of tFhuA via a flexible (GGS)_2_ tether. In addition, we fused Adnectin1,^39^ a 94-residue monobody, a recombinant protein based on the fibronectin type-III (FN3) domain,^49^ to tFhuA in the same way as individual affibodies. The molecular weight of the membrane proteins closely centers around 60 kDa and within the range of 57–63 kDa (Table S5 and Figure S1). In addition, we opportunistically produced two synthetic membrane proteins by substituting the small antibody-mimetic protein scaffold against EGFR with a native growth factor, either EGF or TGFα.^50^ These native binders likely sample a different interaction site than those probed by the monobody and affibody (see below).

Using the available X-ray crystal structures of FhuA^19^ and the complexes Z_HER2_–HER2 (Figure 2a),^38^ Adnectin1–EGFR (Figure 2b),^39^ EGF–EGFR (Figure 2c),^51^ and TGFα–EGFR (Figure 2d),^41^ we represented the binder-equipped membrane protein–protein complexes in each case, respectively (Figure 2). On the other hand, the water-soluble ligands feature molecular weights between 69 and 72 kDa (Table S6 and Figure S1). Various levels of glycosylation determine the higher apparent molecular weights of EGFR, HER2, and HER3 (see below).^25^ Both protein ligands and membrane receptors are characterized by distinct stretches of hydrophobic and hydrophilic domains, as revealed by hydrophilicity maps (Figures S2 and S3). To confirm protein folding and structural stability of the membrane proteins, we utilized circular dichroism (CD) spectroscopy to identify far-UV spectra and temperature-induced melting curves.^28,30^ The far-UV scans showed the formation of β-barrel structures, as expected with the tFhuA stem, after the slow-dialysis removal of urea for each membrane receptor (Table S7 and Figures S4a–S4g). Once the urea concentration was in the low-millimolar range, the membrane proteins reached their final folded state of a β-barrel. In addition, no significant structural changes were observed at lower urea concentrations. As expected, the exchange of different binders did not significantly impact the molar ellipticity, [θ], of each membrane protein because each protein structure was made mostly of the same tFhuA stem. Furthermore, we conducted CD melting curves, which indicated a relatively stable folded structure for all membrane proteins over a broad temperature range. The effective melting temperature of these proteins was ∼70 °C (see Tables S8 and S9, as well as Figure S4h). This result is in accordance with prior differential scanning calorimetry determinations that showed a melting transition of 65 °C for the β-barrel domain of FhuA.^52^

**Figure 2:**
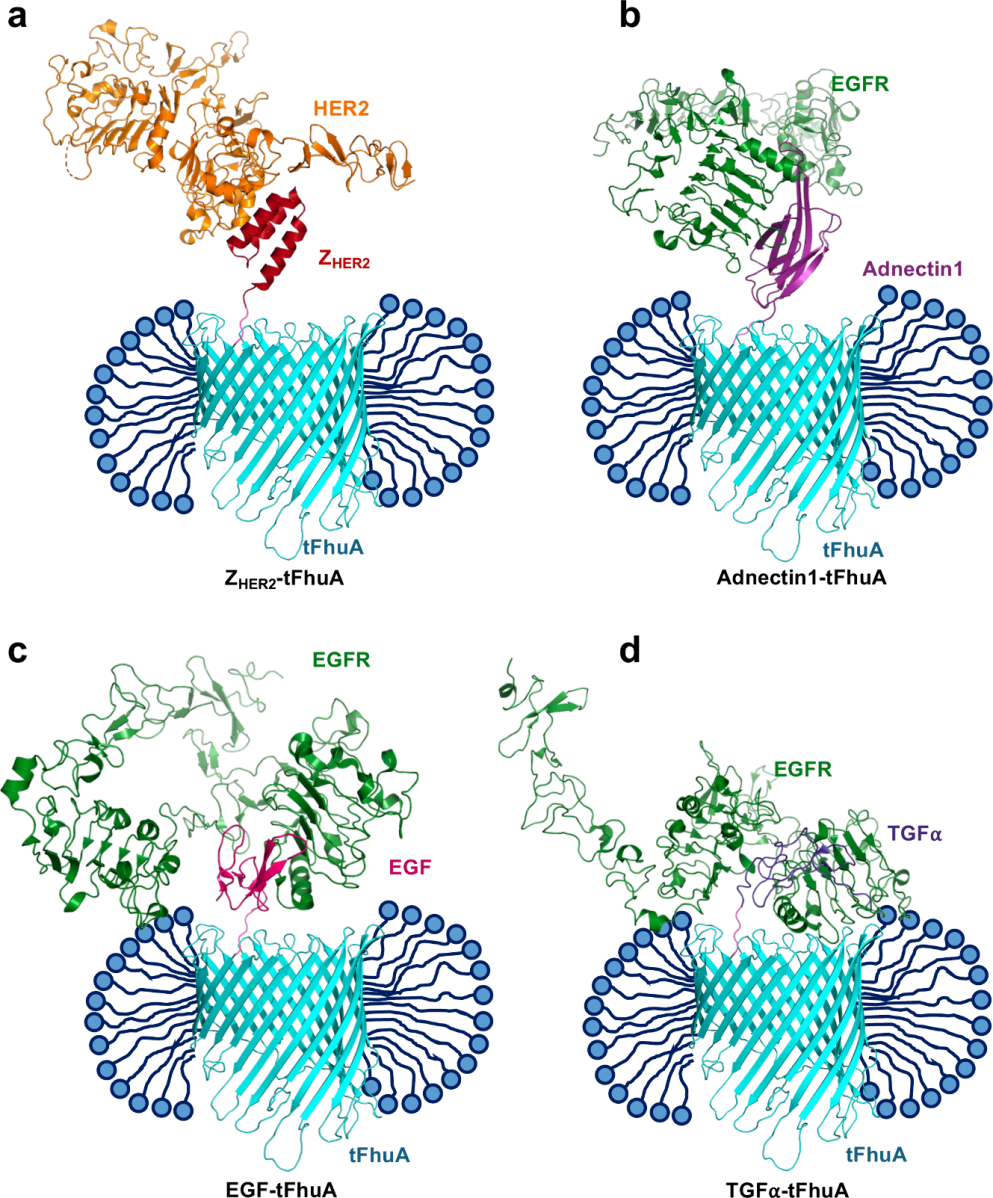
Three-dimensional structures of the membrane receptor–protein ligand complexes. (a) The molecular model cartoon of the complex made by Z_HER2_-tFhuA (red-cyan) with HER2 (orange) using the structure of Z_HER2_–HER2 (3MZW.pdb).^38^ (b) The molecular model cartoon of the complex made by Adnectin1-tFhuA (magenta-cyan) with EGFR (green) using the structure of Adnectin1-EGFR (3QWQ.pdb).^39^ (c) The molecular model cartoon of the complex made by EGF-tFhuA (magenta-cyan) with EGFR (green) using the structure of EGF-EGFR (1NQL.pdb).^40^ (d) The molecular model cartoon of the complex made by TGFα-tFhuA (navy-cyan) with EGFR (green) using the structure of TGFα-EGFR (7SZ7.pdb).^41^ The Z_HER2_-tFhuA membrane protein is a single-polypeptide chain protein that comprises a Z_HER2_ affibody (red) fused to the N terminus of tFhuA (1BY3.pdb)^19^ membrane protein stem (cyan) via a flexible (GGS)_2_ tether (pink).

### Determination of the Membrane Protein–Protein Binding Kinetics Using Biolayer Interferometry

Next, we conducted BLI measurements in which the detergent-solubilized synthetic membrane proteins with programmable binders were added to the wells. Here, the protein ligands were chemically attached to the sensor surface via biotin–streptavidin chemistry (see the Experimental Section). These experiments were executed using a 2-fold dilution series of titratable membrane proteins from low- to high-nanomolar concentrations (Figure 3). The association phase was assessed using six concentrations, [*C*], of these membrane proteins. We noted that the BLI response corresponding to the association was amplified in a time-dependent exponential fashion. This finding confirms the bimolecular nature of the complex formation reaction (see the Experimental Section). Next, we pursued negative- and positive-control experiments to validate the specificity of the bimolecular associations of the membrane protein–ligand complexes. In this case, the synthetic membrane proteins either lacked the programmable binder (e.g., only the tFhuA stem) or had a binder that did not match the cognate protein ligand, respectively (Figure S5). In agreement with our expectations, the BLI response was insensitive upon adding higher concentrations of detergent-solubilized membrane proteins in the wells.

**Figure 3:**
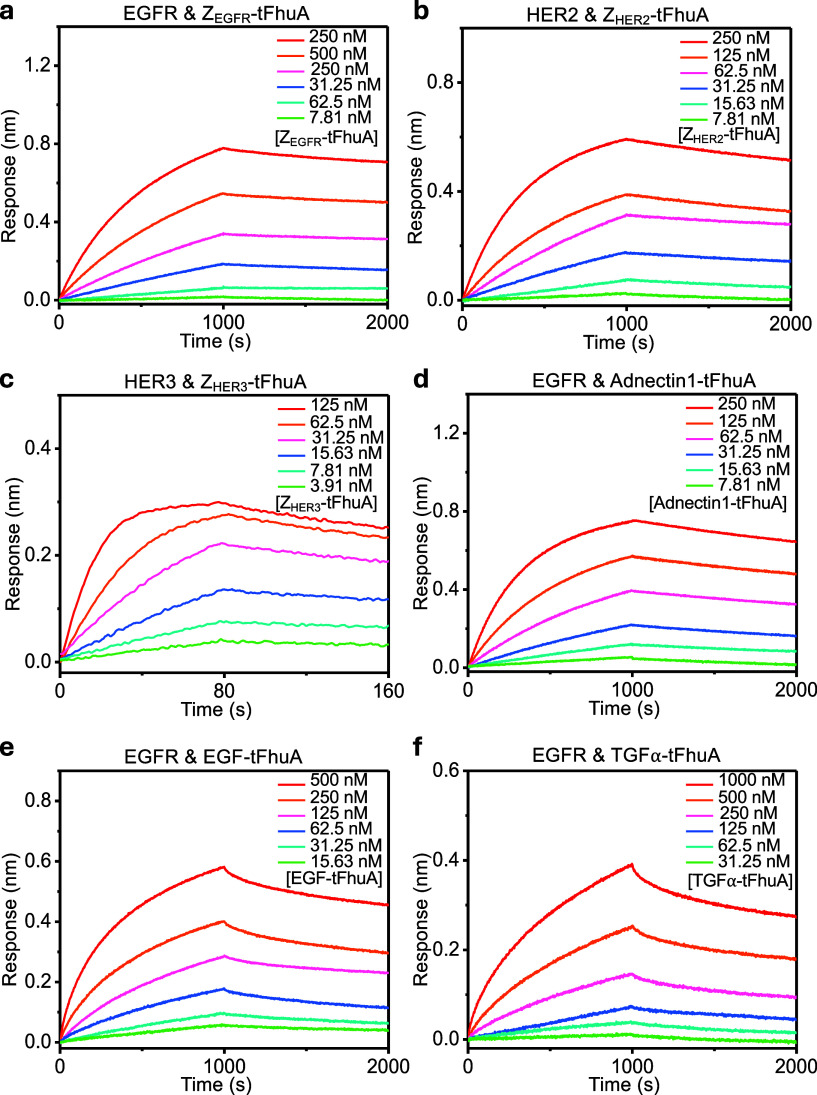
Real-time, label-free BLI experiments show the interaction between a protein ligand attached to the sensor surface and a detergent-solubilized membrane protein: (a) EGFR–Z_EGFR_-tFhuA, (b) HER2–Z_HER2_-tFhuA, (c) HER3–Z_HER3_-tFhuA, (d) EGFR–Adnectin1-tFhuA, (e) EGFR–EGF-tFhuA, and (f) EGFR–TGFα-tFhuA. Here, representative BLI sensorgrams show the association and dissociation phases. For each panel, 50 nM biotinylated ligand was loaded onto SA sensors for 5 min and then dipped into buffers containing six 2-fold serial dilutions of proteomicelles for the association phase. Sensors were then transferred to proteomicelles-free buffer for the dissociation phase.

This BLI response followed a time-dependent exponential amplification, whose magnitude was influenced by the apparent first-order reaction rate constant, *k*_obs_ (see the Experimental Section, eq 1)). This composite kinetic parameter depended on the association (*k*_on_) and dissociation (*k*_off_) rate constants and the effective concentration of proteomicelles in the well, [*C*] (eqs 1–3). Hence, kinetic rate constants *k*_on_ and *k*_off_ were simultaneously determined by recording the dissociation reactions, in which the BLI sensors were placed in proteomicelles-free wells. The dissociation of proteomicelles from the BLI sensor surface, which was indicated by a decline in the sensor response, occurred slowly. Yet, they corresponded to individual initial concentrations of proteomicelles. To quantitatively determine the *k*_on_ and *k*_off_, we utilized global fitting of the multiple BLI response curves corresponding to different concentrations of proteomicelles (see Figures 4a and 4b, as well as Figure S6 and Table S10). This approach was used to indirectly determine the equilibrium dissociation constants, *K*_D_ (see the Experimental Section, eq 4, Figure 4c, and Table S10).

**Figure 4:**
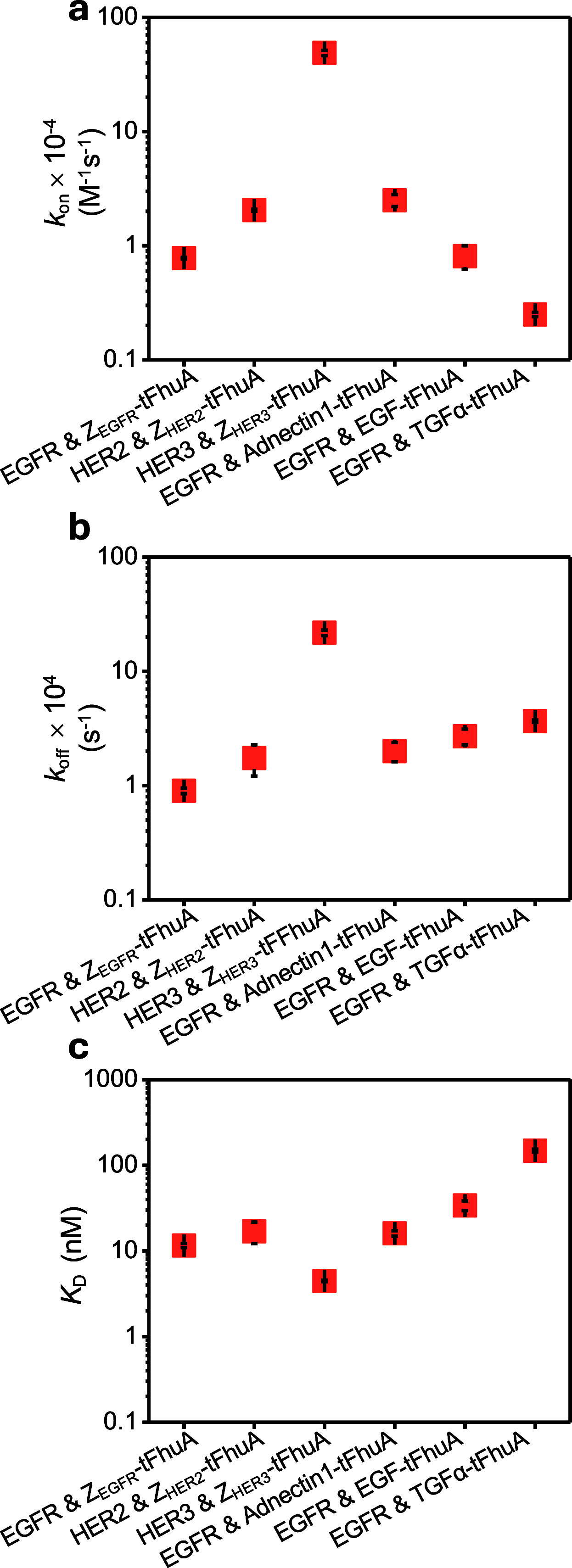
Kinetic rate constants of association and dissociation and the equilibrium dissociation constants of membrane protein–protein ligand interactions. These experiments involved interactions between various protein ligands immobilized onto the BLI sensor surface and free tFhuA-based membrane proteins solubilized in DDM detergent. (a) The kinetic rate constants of association, *k*_on_. (b) The kinetic rate constants of dissociation, *k*_off_. (c) The equilibrium dissociation constants, *K*_D_. Data points represent mean ± standard deviation obtained from *n* = 3 distinct experiments.

The membrane proteins equipped with an antibody-mimetic binder interacted with their cognate protein ligands with a closely similar *k*_on_ in the low 10^4^ M^–1^s^–1^ range, likely due to a high molecular weight of proteomicelles interacting with a protein immobilized onto the sensor surface. The molecular weight of a DDM micelle is ∼70 kDa.^53,54^ Therefore, the molecular weight of a proteomicelle is ∼130 kDa, slightly higher than the apparent molecular weight of ∼100–110 kDa of glycosylated protein ligands (Figure S1). The relatively lower *k*_on_ than the diffusion-limited boundary of the association rate constant^55^ of the protein–protein interactions (∼10^5^ M^–1^s^–1^)^55^ is mainly impacted by the immobilization of one of the binding partners.^56^

For example, a significant increase in the molecular weight of the proteomicelles would result in a slight decrease in the *k*_on_. This is expected, given that *k*_on_ depends on the relative diffusion coefficient of the two interacting partners.^56^ Furthermore, the *k*_on_ value for the binding between HER3 immobilized on the BLI sensor surface and Z_HER3_-tFhuA proteomicelles was approximately 1 order of magnitude higher than that of the others (see Figure 4a, as well as Table S10). From our measurements in this study under various configurations of the immobilized species, alterations in the molecular weight and/or the relative diffusion coefficient of the two interacting partners are accompanied by insignificant changes in the value of *k*_off_. The *k*_off_ values ranged between (0.90 ± 0.05) × 10^–4^ s^–1^ and (21.8 ± 1.2) × 10^–4^ s^–1^ (mean ± standard deviation) for EGFR–Z_EGFR_-tFhuA and HER3–Z_HER3_-tFhuA complex formations, respectively (see Figure 4b, as well as Table S10). Finally, we indirectly inferred the dissociation constant values, *K*_D_, using the kinetic rate constants (*K*_D_ = *k*_off_/*k*_on_; see Figure 4c). The *K*_D_ values for the synthetic proteins equipped with a small antibody mimetic protein scaffold are in the lower nanomolar range, while TGFα-tFhuA exhibited the lowest binding affinity among all synthetic proteins. For example, the *K*_D_ value of EGFR–Z_EGFR_-tFhuA interaction was 16 ± 3 nM. Using an engineered single-molecule nanopore sensor under similar buffer conditions (e.g., 300 mM KCl), we found that the EGFR–Adnectin-1-tFhuA interactions exhibit medium- and high-affinity complexes with the *K*_D_ values of ∼181 nM and ∼34 nM, respectively.^25^ However, in this previous study, EGFR was free in the solution, whereas Adnectin-1-tFhuA was immobilized onto a standing lipid bilayer.

### Quantitative Validation of the Membrane Protein–Protein Binding Kinetics

To validate the high-affinity binding kinetics of membrane protein–protein interactions, we employed closely similar biolayer interferometry measurements when the binder was attached to the BLI sensor surface and the protein ligand was free in the wells (Figure 5). Again, both kinetic rate constants were determined using a related approach to identify the association and dissociation phases (Supplementary Figure S7).

**Figure 5:**
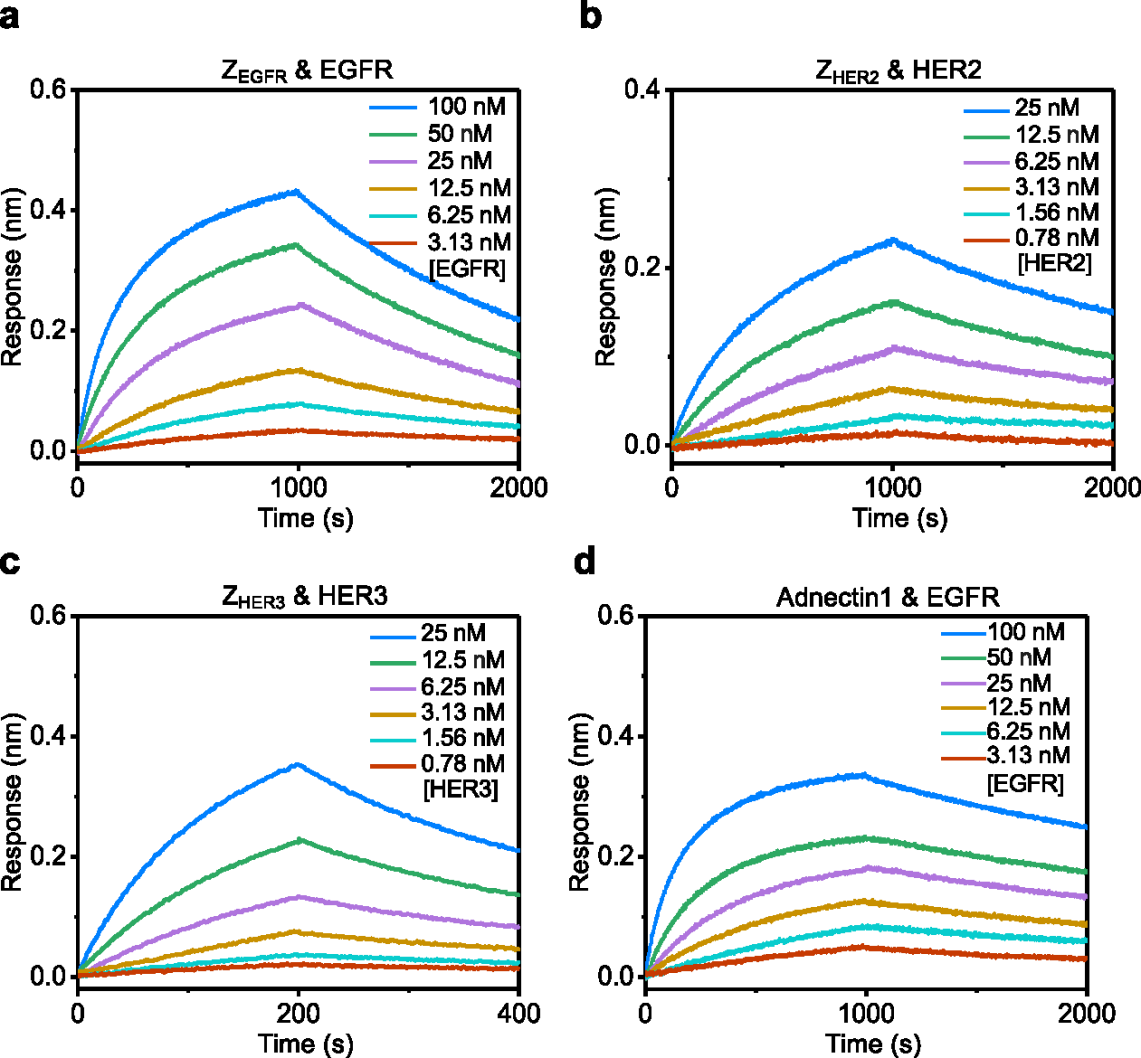
Real-time, label-free BLI experiments of the interaction between a protein binder immobilized onto the sensor surface and its cognate ligand in the well: (a) Z_EGFR_-EGFR, (b) Z_HER2_-HER2, (c) Z_HER3_-HER3, and (d) Adnectin1-EGFR. Here, representative BLI sensorgrams show the association and dissociation phases. For each panel, 50 nM biotin-binder was loaded on streptavidin-coated BLI sensors for 5 min and dipped into the running buffer containing one of the six 2-fold serial dilution concentrations of the protein ligand for the association phase. Sensors were then transferred to a protein ligand-free running buffer for the dissociation phase.

In accordance with the outcomes from the previously determined datasets, the *k*_on_ and *k*_off_ values are in the order of 10^4^ M^–1^s^–1^ and 10^–4^ s^–1^, respectively, confirming the long interaction time between the antibody mimetic binder and its cognate protein ligand (see Table S11 and Figure S8). They correspond to high-affinity binder-ligand complexes in the low nanomolar concentrations. Other researchers have also reported high-affinity interactions of these proteins in determinations that have employed SPR and different buffers, salt concentrations, and immobilization techniques (Tables S12–S14).^39,47,48,57^ The excellent agreement between kinetic and equilibrium determinations when the ligand and binder are immobilized onto the BLI sensor surface can be explained by the closely related molecular mass of the diffusing interacting partner (e.g., proteomicelles versus glycosylated protein ligands; see Tables S5 and S6 and Figure S1). A secondary way to confirm these measurements using BLI is to examine the ligand–binder interactions with the binder freely dispersed in the solution. Unfortunately, we were unable to detect a satisfactory BLI response due to the relatively low molecular mass of the binders, which is 11 kDa or less (Figure S9). This is near the low-molecular-mass detection limit of the analytes by this approach.^7^

However, SPR^31,36,37^ does not have this shortcoming, so we conducted these measurements as an alternative validation using a different binding platform. Here, the binder was injected into the running buffer while the protein ligand was attached onto the sensor surface (see the Experimental Section, as well as Figure S10 and Table S15). Interestingly, in this case, the interactions are somewhat stronger, by up to 1 order of magnitude, compared to those of BLI in which the binder was attached onto the sensor surface (see Tables S11 and S15). These subtle distinctions occurred primarily due to an increase in the rate constants of association, resulting from a slight enhancement in the relative diffusion coefficient of the two interacting partners.^56^ These changes were produced by a high-mobility binder in solution due to its relatively lower molecular mass, compared to that of the ligands. At the same time, the rate constants of dissociation remained unchanged, confirming the validity of the BLI experiments under both configurations (i.e., with and without proteomicelles in the wells).

Finally, we conducted binding interaction studies using SPR when the proteomicelles were injected into the running buffer, under identical conditions to those of the BLI studies discussed above. Again, these data consistently indicate high-affinity interactions between the membrane protein receptors and their cognate protein ligands (see Figures S11 and S12 and Table S16). It should be noted that executing binding experiments with proteomicelles in the microfluidic system of the SPR instrument is more challenging, due to the high sensitivity of this approach to potential clogging and nonspecific adsorption on the sensor surface. This experimental shortcoming occasionally results in a significantly slower, but artifactual dissociation phase.

## CONCLUDING REMARKS

It is generally accepted that membrane proteins represent the most common targets for drug candidates, sparking persistent interest in therapeutics. While the detailed real-time kinetics of membrane protein–ligand interactions are paramount, there are limited experimental opportunities for their robust, accurate, and scalable determination. In this article, we demonstrate that pre-equilibrium kinetic measurements of an antibody mimetic-containing membrane protein in conjunction with its cognate protein ligand can be performed using BLI. While other methods require technical adaptations and lengthy times for calibration and optimization, this experimental design should be readily used without further purification steps to control protein-free detergent structures (e.g., micelles). In addition, these determinations are amenable to parallel recordings of large sample sizes. It is essential to note that BLI is a clog-, microfluidics-, and mass-transfer-free optical platform, and this method can be utilized without the need for membrane protein immobilization on the sensor surface. Finally, this approach can be applied to measurements with challenging heterogeneous solutions, which are otherwise problematic to integrate with prevailing methods due to a significant deterioration in the signal-to-noise ratio.

## Supplementary Material



## Notes

List of primers used in this work; amino acid sequences of proteins used in this study; PDB entries used to visualize all protein structures employed in this study; physicochemical properties of the synthetic membrane proteins used in this study; circular dichroism experiments for confirming the native conformation of the synthetic membrane proteins; negative- and positive-controls BLI measurements for membrane protein–protein interaction; the association (*k*_on_) and dissociation (*k*_off_) rate constants and equilibrium dissociation constant (*K*_D_) of membrane protein–protein ligand interactions were determined by biolayer interferometry (BLI) measurements; equilibrium dissociation constants (*K*_D_) of binder–protein ligand interactions reported by other research groups under different experimental circumstances; BLI tests with the small proteins immobilized onto the sensor surface show no response; surface plasmon resonance (SPR) experiments as confirmatory tests of the BLI measurements; supplementary references (PDF)
