# Supplementary material for: Real-Time Binding Kinetics of Membrane Protein–Protein Interactions in a Membraneless Setting
Source: Anal Chem. 2025 Oct 27;97(44):24849–58. doi: 10.1021/acs.analchem.5c05510 (PMC12613146; doi:10.1021/acs.analchem.5c05510)
Supplement: Supplementary file 1 [file ac5c05510_si_001.pdf]

SUPPORTING INFORMATION FILE

Real-time binding kinetics of membrane protein-protein interactions in a membraneless setting

Yazheng Wang[1,2], Yalun Wu[3], Lauren A. Mayse[1,4], Danny Capucilli[3], Po-Jung J. Huang[3], Sekar Ramachandran[3], Soching Luikham[1], Jeung-Hoi Ha[5], Stewart N. Loh[5], Aaron J. Wolfe[1,3,6], and Liviu Movileanu[1,2,4,7*]

[1]*Department of Physics, Syracuse University, 201 Physics Building, Syracuse, New York 13244, USA*

[2]*Department of Biomedical and Chemical Engineering, Syracuse University, 329 Link Hall, Syracuse, New York 13244, USA*

[3]*Ichor Life Sciences, Inc., 831 James Street, Syracuse, New York 13203, USA*

[4]*The BioInspired Institute, Syracuse University, Syracuse, New York, 13244, USA*

[5]*Department of Biochemistry and Molecular Biology, State University of New York - Upstate Medical University, 4249 Weiskotten Hall, 766 Irving Avenue, Syracuse, New York 13210, USA*

[6]*Department of Chemistry, State University of New York, College of Environmental Science andForestry, 1 Forestry Dr., Syracuse, New York 13210, USA*

[7]*Department of Biology, Syracuse University, 114 Life Sciences Complex, Syracuse, New York 13244, USA*

Keywords: FhuA; Biolayer Interferometry; Membrane Protein Design; Protein-Protein Interactions.

*The corresponding author:
Liviu Movileanu, PhD, Phone: 315-443-8078; E-mail: lmovilea@syr.edu

Table of Contents

1-List of primers used in this work……………………………………………………….S3

2. Amino acid sequences of proteins used in this study……………………………………..S3

3. PDB entries used to visualize all protein structures employed in this study……………….S6

4. Physicochemical properties of the synthetic membrane proteins used in this study……..S7

5. Circular dichroism experiments for confirming the native conformation of the synthetic membrane proteins………………………………………………………………………..S11

6. Negative- and positive-controls BLI measurements for membrane protein-protein interaction……………………………………………………………………………………S15

7. The association (k_{on}) and dissociation (k_{off}) rate constants and equilibrium dissociation constant (K_D) of membrane protein-protein ligand interactions were determined by biolayer interferometry (BLI) measurements…………………………………………………………S16

8. Equilibrium dissociation constants (K_D) of binder-protein ligand interactions reported by other research groups under different experimental circumstances………………………………..S21

9. BLI tests with the small proteins immobilized onto the sensor surface show no response..S22

10. Surface plasmon resonance (SPR) experiments as confirmatory tests of the BLI measurements………………………………………………………………………………S23

11. Supplementary references………………………………………………………………S27

1. List of primers used in this study.

Supplementary Table S1. List of primers used in this study.

Primer name	Sequences (5´-3´)
Z_{EGFR}tFhuA _forward	GAACGGCTGGCAGATGACCGCGTTTATTGCGAGCCTGGTGGATGA CCCCTCGCAGAGC
Z_{EGFR}tFhuA _reverse	AGGTTCGGCAGGTTACGAATTTCTTCCCACGCCGCCCACATCTCTT TATTAAACTTGTTATCCACGGTAC
Z_{EGFR}_forward	CATCATCATTAACACCACCACCACCACCACTGAGATC
Z_{EGFR}_reverse	ATGATGATGGCTGCCGCCGGATCCGCC
Z_{HER2}_forward	CATCATCATTAACACCACCACCACCACCACTGAGATC
Z_{HER2}_reverse	ATGATGATGGCTGCCGCCGGATCCGCC
Z_{HE3}_forward	GGCGGCAGCCATCATCATCATCATCATTAACACCAC
Z_{HER3}_reverse	GGATCCGCCTTTCGGGGCCTGTGCATC

2. Amino acid sequences of proteins used in this study.

Supplementary Table S2. Synthetic membrane protein sequences were used in this work. This table shows the sequence of our protein nanopore sensor, Z(GGS)$_2$tFhuA. Here, Z is an affibody (binder), (GGS)$_2$ is a flexible tether, and t-FhuA is a transducer protein.

**Protein sequence for Z_{EGFR} (GGS)$_2$ tFhuA**
MVDNKFNKEMWAAWEEIRNLPNLNGWQMTAFIASLVDDPSQSANLLAEAKKLNDAQ APKGGSGGSLKEVQFKAGTDSLFQTGFDFSDSLDDDGVYSYRLTGLARSANAQQKGS EEQRYAIAPAFTWRPDDKTNFTFLSYFQNEPETGNSEGSTYSRNEKMVGYSFDHEFND TFTVRQNLRFAENKTSQNSVYGNSEGSRKYVVDDEKLQNFSVDTQLQSKFATGDIDH TLLTGVDFMRMRNDINAWFGYNSEGSSGPYRILNKQKQTGVYVQDQAQWDKVLVTL GGRYDWADQESLNRVAGTTDKRDDKQFTWRGGVNYLFDNGVTPYFSYSESFEPSSQV GKDGNIFAPSKGKQYEVGVKYVPEDRPIVVTGAVYNLTKTNNLMADPEGSFFSVEGG EIRARGVEIEAKAALSASVNVVGSYTYTDAEYTTDTTYKGNTPAQVPKHMASLWADY TFFDGPLSGLTLGTGGRYTNSEGSYTVVDALVRYDLARVGMAGSNVALHVNSEGSQV VATATFRF

Protein sequence for Z_{HER2}(GGS)$_2$tFhuA

MVDNKFNKEMRNAYWEIALLPNLNNQQKRAFIRSLYDDPSQSANLLAEAKKLNDAQ
APKGGSGGSLKEVQFKAGTDSLFQTGFDFSDSLDDDGVYSYRLTGLARSANAQQKGS
EEQRYAIAPAFTWRPDDKTNFTFLSYFQNEPETGNSEGSTYSRNEKMVGYSFDHEFND
TFTVRQNLRFAENKTSQNSVYGNSEGSRKYVVDDEKLQNFSVDTQLQSKFATGDIDH
TLLTGVDFMRMRNDINAWFGYNSEGSSGPYRILNKQKQTGVYVQDQAQWDKVLVTL
GGRYDWADQESLNRVAGTTDKRDDKQFTWRGGVNYLFDNGVTPYFSYSESFEPSSQV
GKDGNIFAPSKGKQYEVGVKYVPEDRPIVVTGAVYNLTKTNNLMADPEGSFFSVEGG
EIRARGVEIEAKAALSASVNVVGSYTYTDAEYTTDTTYKGNTPAQVPKHMASLWADY
TFFDGPLSGLTLGTGGRYTNSEGSYTVVDALVRYDLARVGMAGSNVALHVNSEGSQV
VATATFRF

Protein sequence for Z_{HER3}(GGS)$_2$tFhuA

MVDNKFNKERYLAYYEIWQLPNLNRTQKAAFIGSLQDDPSQSANLLAEAKKLNDAQ
APKGGSGGSLKEVQFKAGTDSLFQTGFDFSDSLDDDGVYSYRLTGLARSANAQQKGS
EEQRYAIAPAFTWRPDDKTNFTFLSYFQNEPETGNSEGSTYSRNEKMVGYSFDHEFND
TFTVRQNLRFAENKTSQNSVYGNSEGSRKYVVDDEKLQNFSVDTQLQSKFATGDIDH
TLLTGVDFMRMRNDINAWFGYNSEGSSGPYRILNKQKQTGVYVQDQAQWDKVLVTL
GGRYDWADQESLNRVAGTTDKRDDKQFTWRGGVNYLFDNGVTPYFSYSESFEPSSQV
GKDGNIFAPSKGKQYEVGVKYVPEDRPIVVTGAVYNLTKTNNLMADPEGSFFSVEGG
EIRARGVEIEAKAALSASVNVVGSYTYTDAEYTTDTTYKGNTPAQVPKHMASLWADY
TFFDGPLSGLTLGTGGRYTNSEGSYTVVDALVRYDLARVGMAGSNVALHVNSEGSQV
VATATFRF

Protein sequence for Adnectin1(GGS)$_2$ tFhuA

MGVSDVPRDLEVVAATPTSLLISWDSGRGSYQYYRITYGETGGNSPVQEFTVPGPVHT
ATISGLKPGVDYTITVYAVTDHKPHADGPHTYHESPISINYRTEIDKPSQGGSGGSLKEV
QFKAGTDSLFQTGFDFSDSLDDDGVYSYRLTGLARSANAQQKGSEEQRYAIAPAFTWR
PDDKTNFTFLSYFQNEPETGNSEGSTYSRNEKMVGYSFDHEFNDTFTVRQNLRFAENK
TSQNSVYGNSEGSRKYVVDDEKLQNFSVDTQLQSKFATGDIDHTLLTGVDFMRMRND
INAWFGYNSEGSSGPYRILNKQKQTGVYVQDQAQWDKVLVTLGGRYDWADQESLNR
VAGTTDKRDDKQFTWRGGVNYLFDNGVTPYFSYSESFEPSSQVGKDGNIFAPSKGKQ
YEVGVKYVPEDRPIVVTGAVYNLTKTNNLMADPEGSFFSVEGGEIRARGVEIEAKAAL
SASVNVVGSYTYTDAEYTTDTTYKGNTPAQVPKHMASLWADYTFFDGPLSGLTLGTG
GRYTNSEGSYTVVDALVRYDLARVGMAGSNVALHVNSEGSQVVATATFRF

Protein sequence for EGF (GGS)$_2$ tFhuA

MNSDSECPLSHDGYCLHDGVCMYIEALDKYACNCVVGYIGERCQYRDLKWWEGGS
GGSLKEVQFKAGTDSLFQTGFDFSDSLDDDGVYSYRLTGLARSANAQQKGSEEQRYAI
APAFTWRPDDKTNFTFLSYFQNEPETGNSEGSTYSRNEKMVGYSFDHEFNDTFTVRQ
NLRFAENKTSQNSVYGNSEGSRKYVVDDEKLQNFSVDTQLQSKFATGDIDHTLLTGV
DFMRMRNDINAWFGYNSEGSSGPYRILNKQKQTGVYVQDQAQWDKVLVTLGGRYD
WADQESLNRVAGTTDKRDDKQFTWRGGVNYLFDNGVTPYFSYSESFEPSSQVGKDG
NIFAPSKGKQYEVGVKYVPEDRPIVVTGAVYNLTKTNNLMADPEGSFFSVEGGEIRAR
GVEIEAKAALSASVNVVGSYTYTDAEYTTDTTYKGNTPAQVPKHMASLWADYTFFD

GPLSGLTLGTGGRYTNSEGSYTVVDALVRYDLARVGMAGSNVALHVNSEGSQVVATA<br>TFRF
**Protein sequence for TGF-α(GGS)$_2$ tFhuA**
MENSTSPLSADPPVAAAVVSHFNDCPDSHTQFCFHGTCRFLVQEDKPACVCHSGYVGA<br>RCEHADLLAVVAASQKKQGGSGGSLKEVQFKAGTDSLFQTGFDFSDSLDDDGVYSYR<br>LTGLARSANAQQKGSEEQRYAIAPAFTWRPDDKTNFTFLSYFQNEPETGNSEGSTYSR<br>NEKMVGYSFDHEFNDTFTVRQNLRFAENKTSQNSVYGNSEGSRKYVVDDEKLQNFS<br>VDTQLQSKFATGDIDHTLLTGVDFMRMRNDINAWFGYNSEGSSGPYRILNKQKQTGV<br>YVQDQAQWDKVLVTLGGRYDWADQESLNRVAGTTDKRDDKQFTWRGGVNYLFDN<br>GVTPYFSYSESFEPSSQVGKDGNIFAPSKGKQYEVGVKYVPEDRPIVVTGAVYNLTKT<br>NNLMADPEGSFFSVEGGEIRARGVEIEAKAALSASVNVVGSYTYTDAEYTTDTTYKG<br>NTPAQVPKHMASLWADYTFFDGPLSGLTLGTGGRYTNSEGSYTVVDALVRYDLARVG<br>MAGSNVALHVNSEGSQVVATATFRF

<u>**Supplementary Table S3.**</u> **The amino acid sequence of water-soluble protein ligands used in this work.** They are the ectodomains (e.g., extracellular domains) of the human epidermal growth factor receptors 1 (HER1/EGFR), 2 (HER2), and 3 (HER3). These sequences also include a hexahistidine tag.

**Protein sequence for EGFR$^{25\text{-}645}$**
MVLEEKKVCQGTSNKLTQLGTFEDHFLSLQRMFNNCEVVLGNLEITYVQRNYDLSFL<br>KTIQEVAGYVLIALNTVERIPLENLQIIRGNMYYENSYALAVLSNYDANKTGLKELPMR<br>NLQEILHGAVRFSNNPALCNVESIQWRDIVSSDFLSNMSMDFQNHLGSCQKCDPSCPN<br>GSCWGAGEENCQKLTKIICAQQCSGRCRGKSPSDCCHNQCAAGCTGPRESDCLVCRK<br>FRDEATCKDTCPPLMLYNPTTYQMDVNPEGKYSFGATCVKKCPRNYVVTDHGSCVR<br>ACGADSYEMEEDGVRKCKKCEGPCRKVCNGIGIGEFKDSLSINATNIKHFKNCTSISGD<br>LHILPVAFRGDSFTHTPPLDPQELDILKTVKEITGFLLIQAWPENRTDLHAFENLEIIRGR<br>TKQHGQFSLAVVSLNITSLGLRSLKEISDGDVIISGNKNLCYANTINWKKLFGTSGQKT<br>KIISNRGENSCKATGQVCHALCSPEGCWGPEPRDCVSCRNVSRGRECVDKCNLLEGEP<br>REFVENSECIQCHPECLPQAMNITCTGRGPDNCIQCAHYIDGPHCVKTCPAGVMGENN<br>TLVWKYADAGHVCHLCHPNCTYGCTGPGLEGCPTNGPKIPSHHHHHH
**Protein sequence for HER2$^{1\text{-}652}$**
MELAALCRWGLLLALLPPGAASTQVCTGTDMKLRLPASPETHLDMLRHLYQGCQVV<br>QGNLELTYLPTNASLSFLQDIQEVQGYVLIAHNQVRQVPLQRLRIVRGTQLFEDNYAL<br>AVLDNGDPLNNTTPVTGASPGGLRELQLRSLTEILKGGVLIQRNPQLCYQDTILWKDIF<br>HKNNQLALTLIDTNRSRACHPCSPMCKGSRCWGESSEDCQSLTRTVCAGGCARCKGP<br>LPTDCCHEQCAAGCTGPKHSDCLACLHFNHSGICELHCPALVTYNTDTFESMPNPEGR<br>YTFGASCVTACPYNYLSTDVGSCTLVCPLHNQEVTAEDGTQRCEKCSKPCARVCYGL<br>GMEHLREVRAVTSANIQEFAGCKKIFGSLAFLPESFDGDPASNTAPLQPEQLQVFETLE<br>EITGYLYISAWPDSLPDLSVFQNLQVIRGRILHNGAYSLTLQGLGISWLGLRSLRELGS<br>GLALIHHNTHLCFVHTVPWDQLFRNPHQALLHTANRPEDECVGEGLACHQLCARGH<br>CWGPGPTQCVNCSQFLRGQECVEECRVLQGLPREYVNARHCLPCHPECQPQNGSVTC

FGPEADQCVACAHYKDPPFCVARCPSGVKPDLSYMPIWKFPDEEGACQPCPINCTHSC VDLDDKGCPAEQRASPLTHHHHHH
**Protein sequence for HER3$^{1\text{-}643}$**
MRANDALQVLGLLFSLARGSEVGNSQAVCPGTLNGLSVTGDAENQYQTLYKLYERC EVVMGNLEIVLTGHNADLSFLQWIREVTGYVLVAMNEFSTLPLPNLRVVRGTQVYDG KFAIFVMLNYNTNSSHALRQLRLTQLTEILSGGVYIEKNDKLCHMDTIDWRDIVRDRD AEIVVKDNGRSCPPCHEVCKGRCWGPGSEDCQTLTKTICAPQCNGHCFGPNPNQCCH DECAGGCSGPQDTDCFACRHFNDSGACVPRCPQPLVYNKLTFQLEPNPHTKYQYGGV CVASCPHNFVVDQTSCVRACPPDKMEVDKNGLKMCEPCGGLCPKACEGTGSGSRFQ TVDSSNIDGFVNCTKILGNLDFLITGLNGDPWHKIPALDPEKLNVFRTVREITGYLNIQS WPPHMHNFSVFSNLTTIGGRSLYNRGFSLLIMKNLNVTSLGFRSLKEISAGRIYISANR QLCYHHSLNWTKVLRGPTEERLDIKHNRPRRDCVAEGKVCDPLCSSGGCWGPGPGQ CLSCRNYSRGGVCVTHCNFLNGEPREFAHEAECFSCHPECQPMEGTATCNGSGSDTC AQCAHFRDGPHCVSSCPHGVLGAKGPIYKYPDVQNECRPCHENCTQGCKGPELQDCL GQTLVLIGKTHLTHHHHHH

3. PDB entries used to visualize all protein structures employed in this study.

Supplementary Table S4. ***PDB entry files were used to visualize all protein structures employed in this study.***

Protein/Protein complex	PDB accession Code	Source's URL
FhuA	1BY3[1]	https://www.rcsb.org/structure/1by3
Z_{HER2}-HER2	3MZW[2]	https://www.rcsb.org/structure/3MZW
Adnectin1-EGFR	3QWQ[3]	https://www.rcsb.org/structure/3QWQ
EGF-EGFR	1NQL[4]	https://www.rcsb.org/structure/1NQL
TGFα-EGFR	7SZ7[5]	https://www.rcsb.org/structure/7SZ7

4. Physicochemical properties of the synthetic membrane proteins used in this study.

<u>**Supplementary Table S5.**</u> **Physicochemical properties of the synthetic membrane proteins used in this study.**

Protein	Molecular weight (kDa)	Length (residues)[a]	Charge[b]	Kyte−Doolittle hydropathy index[c]	Length of the binder (residues)[d]	Charge of the binder[b]
tFhuA	50.6	455	-15.0	-277.2	NA[e]	NA[e]
Z_{EFGR}-tFhuA	57.7	520	-17.0	-310.7	59	-2.2
Z_{HER2}-tFhuA	57.8	520	-13.0	-330.9	59	+1.7
Z_{HER3}-tFhuA	57.8	520	-14.0	-327.2	59	+0.7
Adnectin1-tFhuA	62.7	569	-19.6	-330.9	108	-4.8
EGF-tFhuA	57.0	513	-20.4	-300.3	52	-5.6
TGFα-tFhuA	59.1	537	-18.1	-289.4	76	-3.3

[a]The sequences of the polypeptides are presented in **Supplementary Table S2**.
[b]The charge was estimated at pH 7.5.
The Kyte-Doolittle hydropathy index is the sum of the Kyte-Doolittle hydropathy indices of the individual amino acids in the polypeptide.[6, 7]
[d]The protein binder was fused at the N terminus of the tFhuA membrane protein stem via a flexible $(GGS)_2$ tether.[8]
[e]This is a binder-free membrane protein.

<u>**Supplementary Table S6.**</u> **Physicochemical properties of the water-soluble protein ligands used in this study.**

Protein	Molecular weight (kDa)[d]	Length (residues) [a]	Charge[b]	Kyte−Doolittle hydropathy index[c]
EFGR	69.6	629	-6.8	-241.3
HER2	72.4	658	-19.1	-144.7
HER3	71.5	649	-6.2	-219.5

[a]The sequences of the polypeptides are presented in **Supplementary Table S3**.
[b]The charge was estimated at pH 7.5.
[c]The Kyte-Doolittle hydropathy index is the sum of the Kyte-Doolittle hydropathy indices of the individual amino acids in the polypeptide.[6, 7]
[d]This is the predicted molecular weight. However, due to glycosylation, the apparent molecular weight will be higher under reducing conditions due to glycosylation of different Asn residues (**Supplementary Figure S1**).

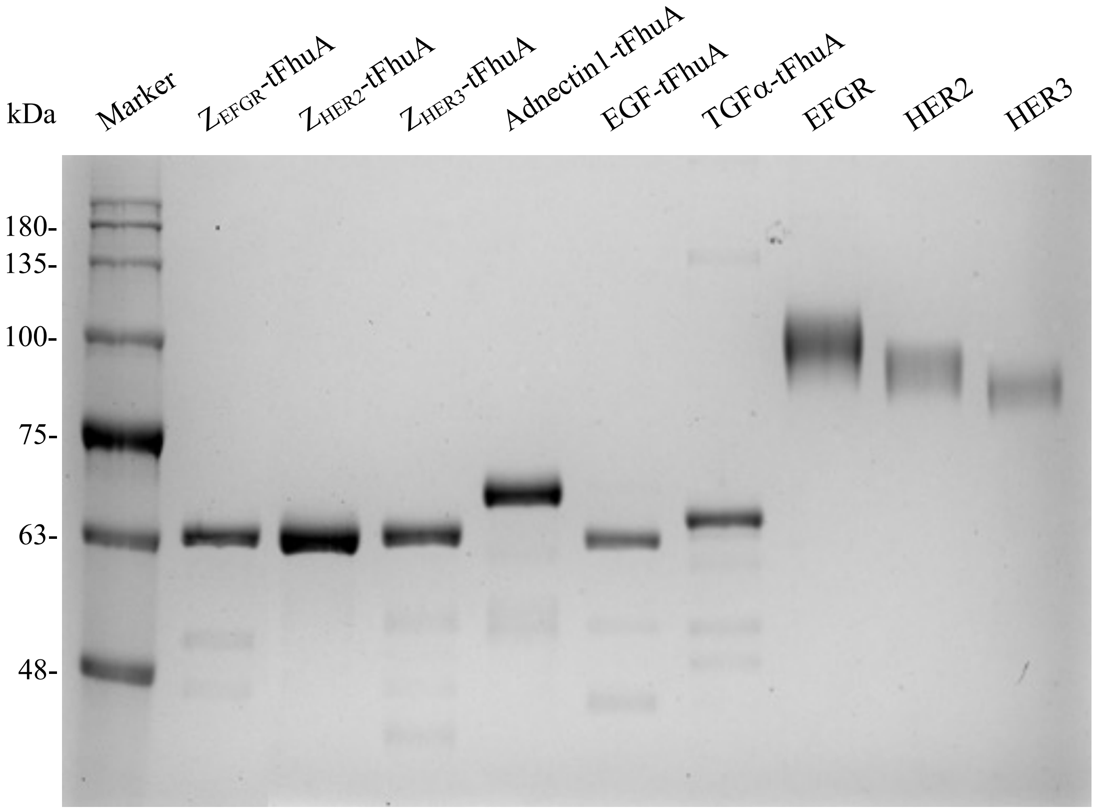

<u>Supplementary Figure S1</u>**. An SDS-PAGE gel analysis of the six synthetic membrane proteins and three water-soluble ligands utilized in this study.** The purity and size of all nine proteins were checked by an 8% SDS-PAGE gel analysis. The expected molecular weights of the synthetic membrane proteins and water-soluble ligands are listed in **Supplementary Table S5** and **Table S6**, respectively. Various levels of glycosylation determine the higher apparent molecular weights of EGFR, HER2, and HER3.[9] For example, the expected molecular weight of EGFR is 69.6 kDa. However, we observed a higher apparent molecular weight due to glycosylation. These water-soluble ligands were purified using a polyhistidine-tag column (**Experimental section**).

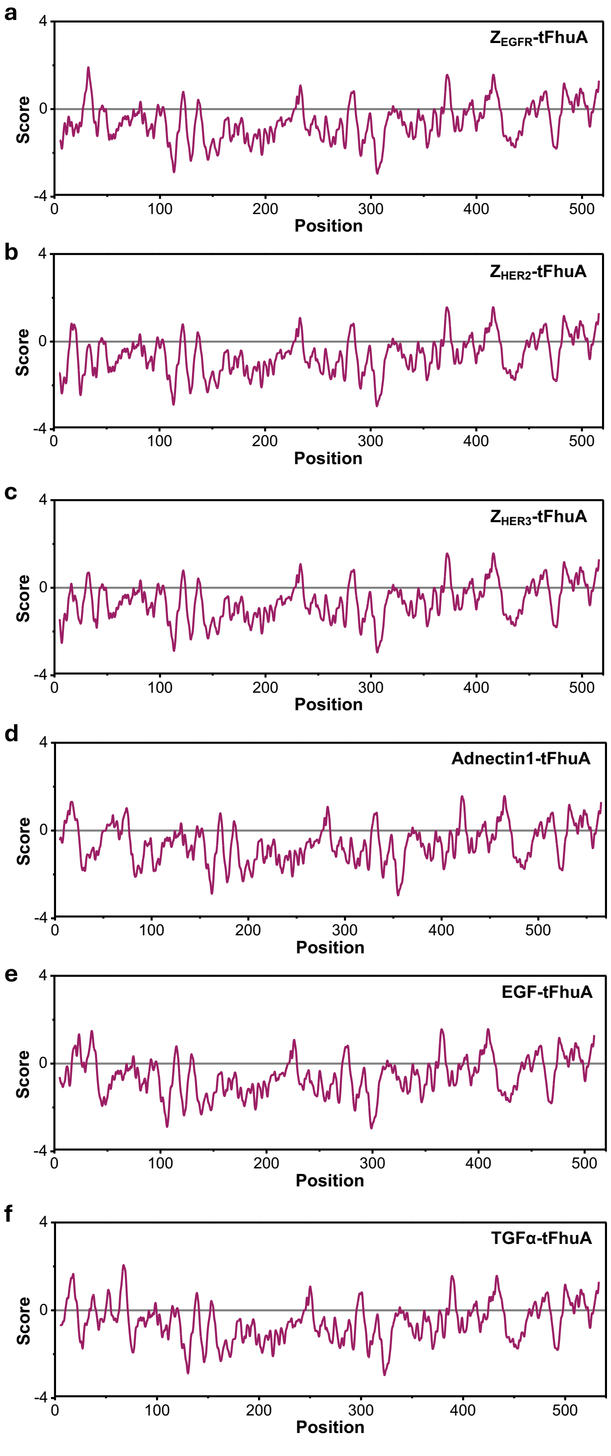

<u>Supplementary Figure S2</u>. Protein hydrophilicity plots for synthetic membrane proteins used in this study. The score is based on the Kyte-Doolittle hydropathy indices of the individual amino acids in the protein sequence.[6, 7] The window span was 9 residues.

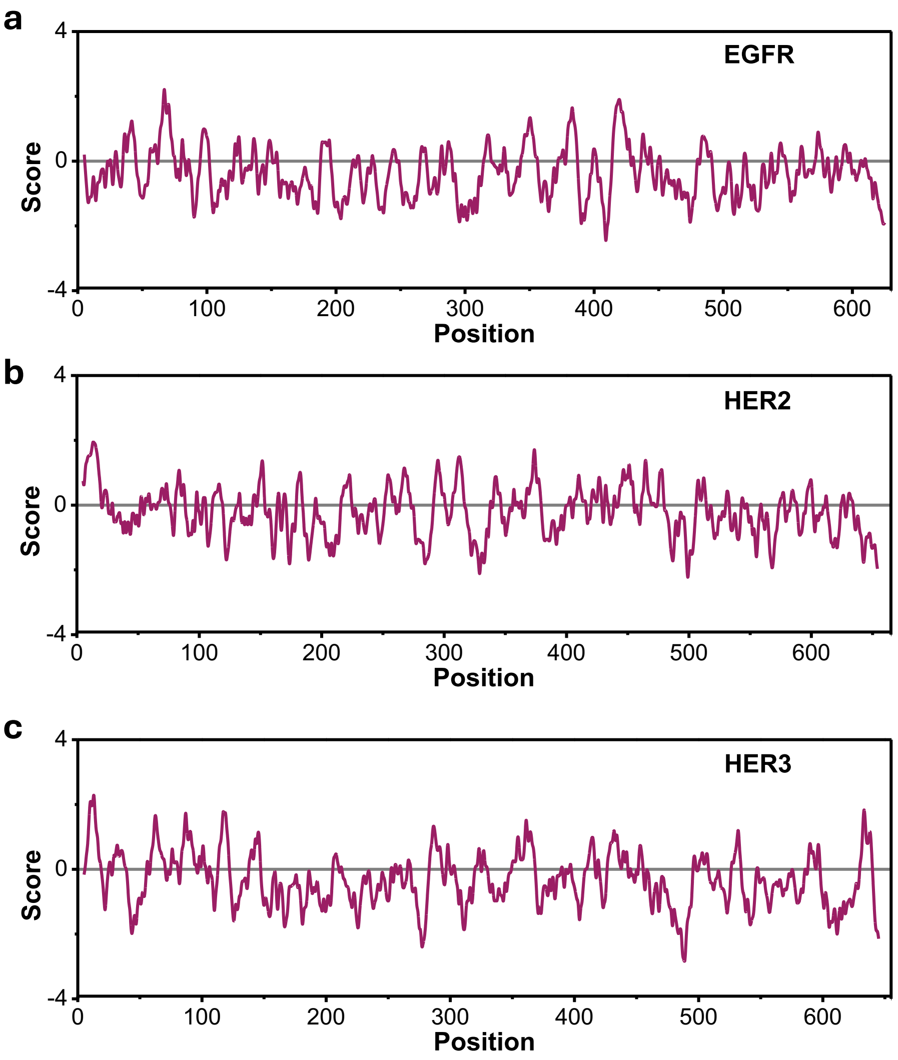

Supplementary Figure S3. Protein hydrophilicity plots for the three hydrophilic proteins used in this study. The score is based on the Kyte-Doolittle hydropathy indices of the individual amino acids in the protein sequence.[6, 7] The window span was 9 residues.

5. Circular dichroism experiments for confirming the native conformation of the synthetic membrane proteins.

Supplementary Table S7. Molar ellipticity, [θ], at 217 nm for all membrane proteins examined in this work. 217 nm was selected.[10] [θ] is reported in deg cm^2/dmol. These measurements were conducted using samples in 200 mM KCl, 20 mM Tris-HCl, pH 7.5, 12 pM urea, at 22°C. Data represent mean ± s.d. acquired with n = 3 independent refolded samples.

Membrane Protein	[θ] (deg cm^2/dmol)
Z_{EGFR}-tFhuA	-7,098 ± 1,360
Z_{HER2}-tFhuA	-7,250 ± 688
Z_{HER3}-tFhuA	-7,973 ± 700
Adnectin1-tFhuA	-8,454 ± 2,028
EGF-tFhuA	-9,105 ± 2,429
TGFα-tFhuA	-6,602 ± 1,181
tFhuA	-5,888 ± 1,126

Supplementary Table S8. Molar ellipticity, [θ], of all proteins examined in this work during a temperature melt experiment. Millidegrees of differential absorption at 219 nm were converted to molar ellipticity, as described in the Experimental Section.[11] [θ] is reported in deg cm^2/dmol. These measurements were conducted using samples in 200 mM KCl, 20 mM Tris-HCl, pH 7.5, and 12 pM urea. At temperatures above 80°C, the observed standard deviation is significant because the protein began to precipitate. This was visible, altering the absorbance and the remaining protein in solution. Data represent mean ± s.d. acquired with n = 3 independent refolded samples.

Protein	20°C [θ] (deg cm^2/dmol) × 10^{-3}	30°C [θ] (deg cm^2/dmol) ×10^{-3}	40°C [θ] (deg cm^2/dmol) × 10^{-3}	50°C [θ] (deg cm^2/dmol) × 10^{-3}	60°C [θ] (deg cm^2/dmol) × 10^{-3}	70°C [θ] (deg cm^2/dmol) × 10^{-3}	80°C [θ] (deg cm^2/dmol) × 10^{-3}	90°C [θ] (deg cm^2/dmol) × 10^{-3}
Z_{EGFR}-tFhuA	-8.9 ± 1.5	-8.4 ± 1.4	-8.4 ± 1.4	-8.1 ± 1.0	-7.9 ± 1.5	-4.3 ± 1.4	-2.6 ± 1.0	-2.4 ± 0.9
Z_{HER2}-tFhuA	-7.8 ± 0.4	-7.8 ± 0.6	-8.4 ± 0.8	-8.2 ± 1.0	-7.6 ± 1.3	-4.0 ± 1.3	-1.9 ± 0.8	-1.5 ± 0.9
Z_{HER3}-tFhuA	-9.5 ± 1.8	-9.5 ± 1.8	-9.4 ± 1.8	-9.1 ±1.8	-8.3 ± 1.5	-5.2 ± 1.4	-2.5 ± 0.5	-2.6 ± 0.2
Adnectin1-tFhuA	-8.0 ± 0.6	-8.0 ± 0.7	-7.9 ± 0.7	-7.8 ± 0.9	-7.4 ± 1.7	-5.6 ± 1.7	-3.6 ± 1.9	-3.7 ± 2.0
EGF-tFhuA	-8.4 ± 2.2	-8.3 ± 2.2	-8.3 ± 2.2	-8.2 ± 2.1	-7.7 ± 2.4	-5.4 ± 1.9	-3.1 ± 1.9	-3.6 ± 2.1
TGFα-tFhuA	-7.4 ± 1.6	-7.3 ± 1.5	-7.3 ± 1.5	-7.3 ± 1.7	-7.0 ± 1.6	-5.5 ± 1.3	-2.8 ± 1.2	-2.2 ± 1.7
tFhuA	-6.3 ± 1.6	-6.1 ± 1.6	-6.1 ± 1.6	-6.1 ± 1.3	-5.8 ± 1.4	-3.2 ± 0.8	-2.0 ± 0.9	-1.6 ± 0.8

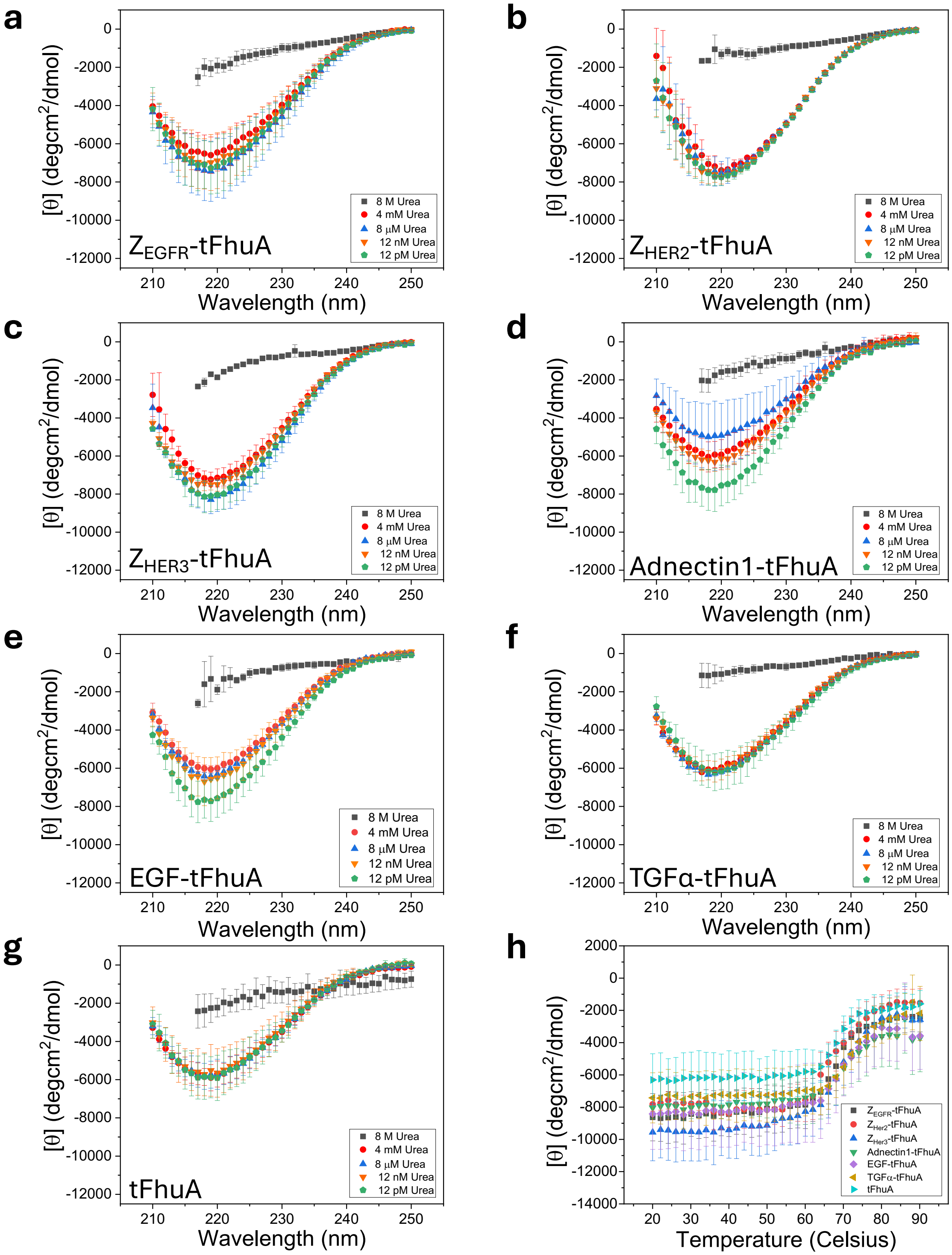

__Supplementary Figure S4.__ **Circular dichroism (CD) spectroscopy reveals the -barrel structure and thermal stability of all expressed membrane proteins.** **(a)** Far-UV CD spectra of Z_{EGFR}-tFhuA, measured from 210 to 250 nm in 200 mM KCl, 20 mM Tris-HCl at pH 7.5, with urea concentrations of 8 M, 4 M, 8 μM, 12 nM, and 12 pM, as labeled. The CD absorption

spectra at 8 M Urea were limited to 215-250 nm due to direct urea absorption. The data shown were derived from three independent refolded samples. **(b)** The same as (a) for Z_{HER2}-tFhuA. **(c)** The same as (a) For Z_{HER3}-tFhuA. **(d)** The same as (a) for Adnectin1-tFhuA. **(e)** The same as (a) for EGF-tFhuA. The data shown for 12 pM urea were derived from two refolded samples; all other data are from three refolded samples. **(f)** The same as (a) for TGFα-tFhuA. The data shown for 4 mM Urea were derived from two refolded samples. **(g)** The same as (a) for tFhuA. The data shown for 8 M urea were derived from two refolded samples. **(h)** Temperature-induced melting curves were performed from 20 to 90°C at 2°C intervals, with a wavelength of 219 nm for all expressed membrane proteins. The data shown were derived from three independent refolded samples. This melting and refolding trajectories were not reversible, as the samples had visible aggregates at 90°C.

Supplementary Table S9. Effective melting temperatures for all membrane proteins. The inflection point on each temperature-dependent curve determines the melting temperature, or where 50% of the molecules are in a single-stranded state and 50% remain in secondary structure. The plots in **Supplementary Figure S4h** were fitted with a Boltzmann sigmoidal curve to extract the melting temperatures of different membrane proteins. Data are provided as mean ± s.e.m. from three independently conducted temperature melt experiments. This set of data agrees with prior differential scanning calorimetry studies of FhuA by Bonhivers and coworkers, who found that the β-barrel domain of FhuA unfolds at 65°C.[12]

**Protein**	**T_m (°C)**
Z_{EGFR}-tFhuA	67.9 ± 0.2
Z_{Her2}-tFhuA	68.5 ± 0.5
Z_{Her3}-tFhuA	68.6 ± 0.4
Adnectin1-tFhuA	70.1 ± 0.7
EGF-tFhuA	69.7 ± 0.4
TGFα-tFhuA	72.5 ± 0.2
tFhuA	68.1 ± 0.1

6. Negative- and positive-control BLI measurements for membrane protein-protein interaction

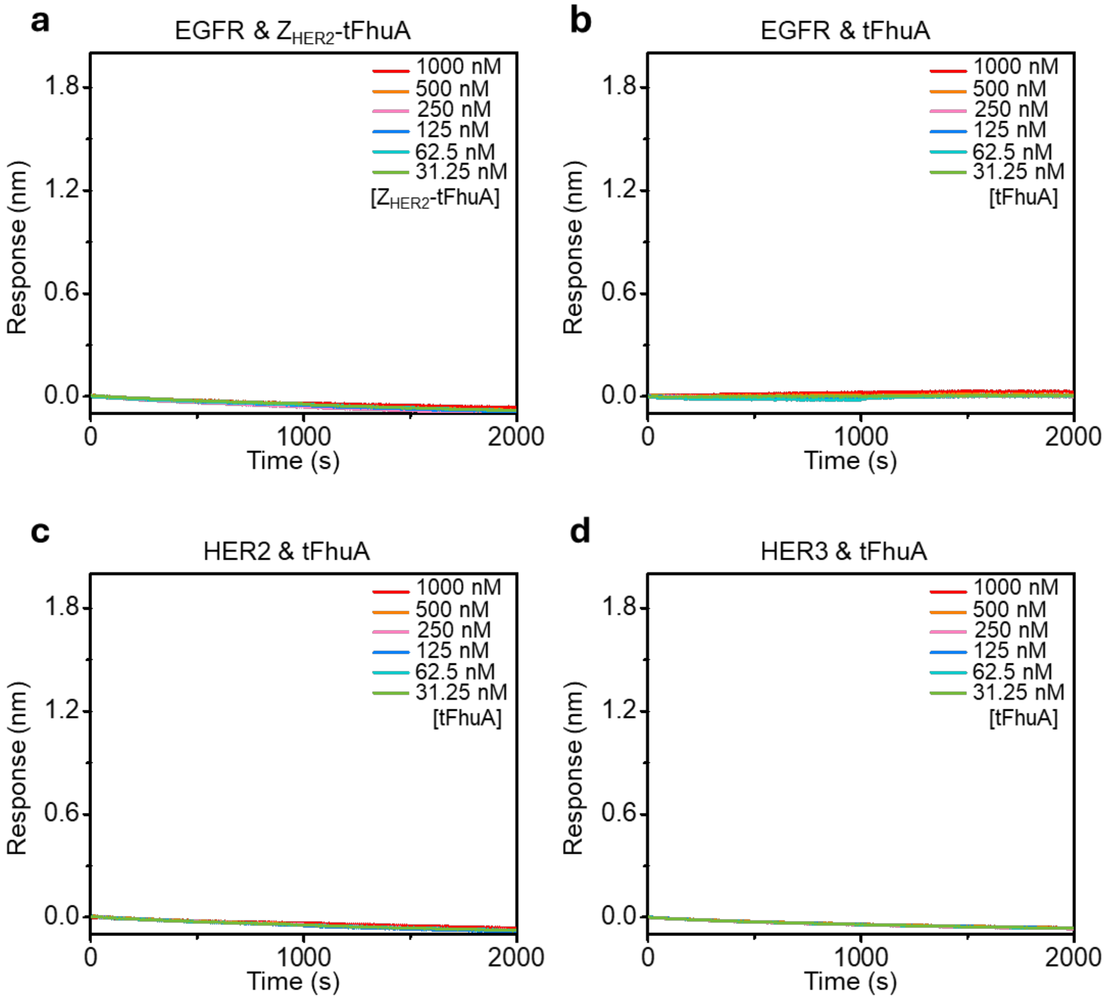

<u>Supplementary Figure S5.</u> BLI sensorgrams of positive- and negative-control experiments. (a) A positive-control family of BLI responses to test the binding of the immobilized EGFR with the free Z_{HER2}-tFhuA-containing proteomicelle. **(b)** A negative-control family of BLI responses to test the binding of EGFR with tFhuA. **(c)** A negative-control family of BLI responses to test the binding of HER2 with tFhuA. **(d)** A negative-control family of BLI responses to test the binding of HER3 with tFhuA. In (a)-(d), 50 nM biotin-labeled ligand was loaded onto streptavidin-coated sensors for 5 min and dipped into buffers containing six two-fold serial dilution concentrations of proteomicelles for the association phase. Individual binding curves are indicated for [tFhuA] from 1000 nM to 31.25 nM. For the dissociation, the sensors were dipped into the proteomicelle-free running buffer. The association and dissociation phases span 0-1000 seconds and 1000-2000 seconds. respectively.

7. The association (k_{on}) and dissociation (k_{off}) rate constants and equilibrium dissociation constant (K_D) of membrane protein-protein ligand interactions were determined by biolayer interferometry (BLI) measurements.

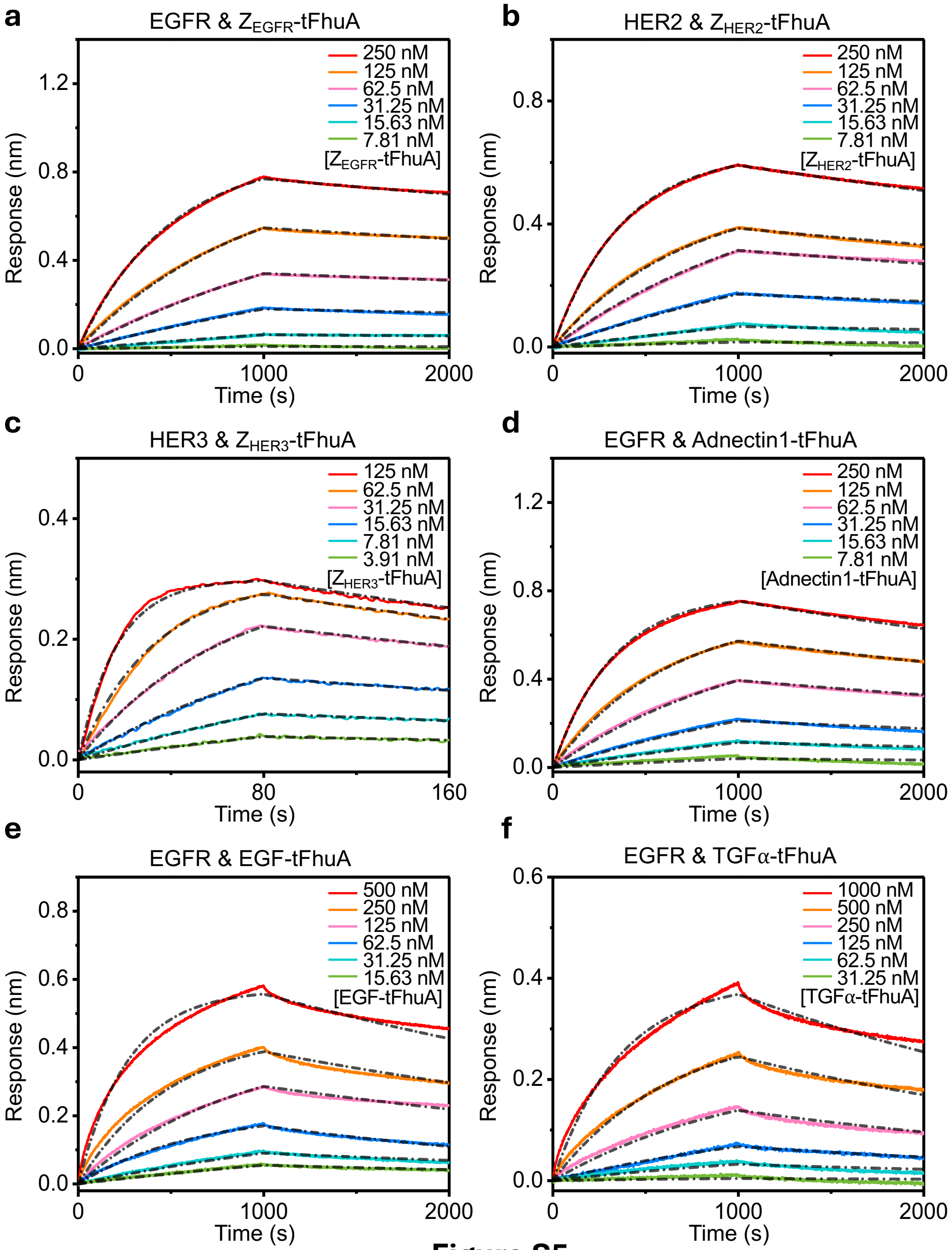

Figure S5

<u>**Supplementary Figure S6.**</u> **Fits of label-free BLI experiments of the membrane protein-protein ligand interactions when the ligand was immobilized onto the BLI sensor surface.** The FortéBio Octet Data Analysis software (FortéBio) was used to fit these binding curves. The dotted black line represents the fitting curve.

<u>Supplementary Table S10.</u> BLI-determined kinetic rate constants of association (k_{on} and k_{off}), and equilibrium dissociation constants (K_D) of the ligand-membrane protein interactions with the membrane proteins added to the wells and the ligands attached to the BLI chip surface. The running buffer included 20 mM Tris-HCl, 300 mM KCl, 0.2% (w/v) DDM, and 1 mg/ml bovine serum albumin (BSA), at pH 7.5. Values of all constants, which were derived using global fits **(Experimental Section)**, indicate mean ± s.e.m. The average values indicate the mean ± s.d., which were acquired using *n* = 3 independent BLI experiments.

	$k_{on} \times 10^{-4}$ ($M^{-1}s^{-1}$)		$k_{off} \times 10^{4}$ (s^{-1})		K_D (nM)	
	Single cycle	Average	Single cycle	Average	Single cycle	Average
EGFR & Z_{EGFR}-tFhuA	0.79 ± 0.01<br>0.79 ± 0.02<br>0.77 ± 0.03	0.78 ± 0.01	0.85 ± 0.01<br>0.95 ± 0.02<br>0.90 ± 0.03	0.90 ± 0.05	10. 90 ± 0.08<br>12.10 ± 0.05<br>11.70 ± 0.01	11.6 ± 0.6
HER2 & Z_{HER2}-tFhuA	2.10 ± 0.01<br>2.02 ± 0.01<br>2.03 ± 0.01	2.05 ± 0.04	2.35 ± 0.01<br>1.50 ± 0.01<br>1.37 ± 0.01	1.74 ± 0.53	11.18 ± 0.04<br>7.43 ± 0.03<br>6.74 ± 0.03	8.5 ± 2.4
HER3 & Z_{HER3}-tFhuA	46.63 ± 0.23<br>48.40 ± 0.28<br>51.60 ± 0.28	48.8 ± 2.52	20.47 ± 0.14<br>22.20 ± 0.16<br>22.80 ± 0.15	21.82 ± 1.21	4.39 ± 0.04<br>4.57 ± 0.04<br>4.41 ± 0.04	4.5 ± 0.1
EGFR & Adnectin1-tFhuA	2.87 ± 0.01<br>2.30 ± 0.01<br>2.33 ± 0.01	2.50 ± 0.32	2.42 ± 0.01<br>1.73 ± 0.01<br>1.82 ± 0.01	1.99 ± 0.38	8.44 ± 0.04<br>7.54 ± 0.03<br>7.82 ± 0.03	7.9 ± 0.5
EGFR & EGF-tFhuA	0.73 ± 0.01<br>0.67 ± 0.01<br>1.02 ± 0.01	0.81 ± 0.19	2.81 ± 0.02<br>2.23 ± 0.03<br>3.06 ± 0.04	2.70 ± 0.43	38.50 ± 0.27<br>33.40 ± 0.17<br>29.93 ± 0.37	33.9 ± 4.3
EGFR & TGFα-tFhuA	0.26 ± 0.01<br>0.24 ± 0.01<br>0.24 ± 0.01	0.25 ± 0.01	3.75 ± 0.03<br>3.57 ± 0.04<br>3.70 ± 0.02	3.67 ± 0.09	144.4 ± 1.7<br>146.7 ± 1.7<br>154.9 ± 1.1	149 ± 6

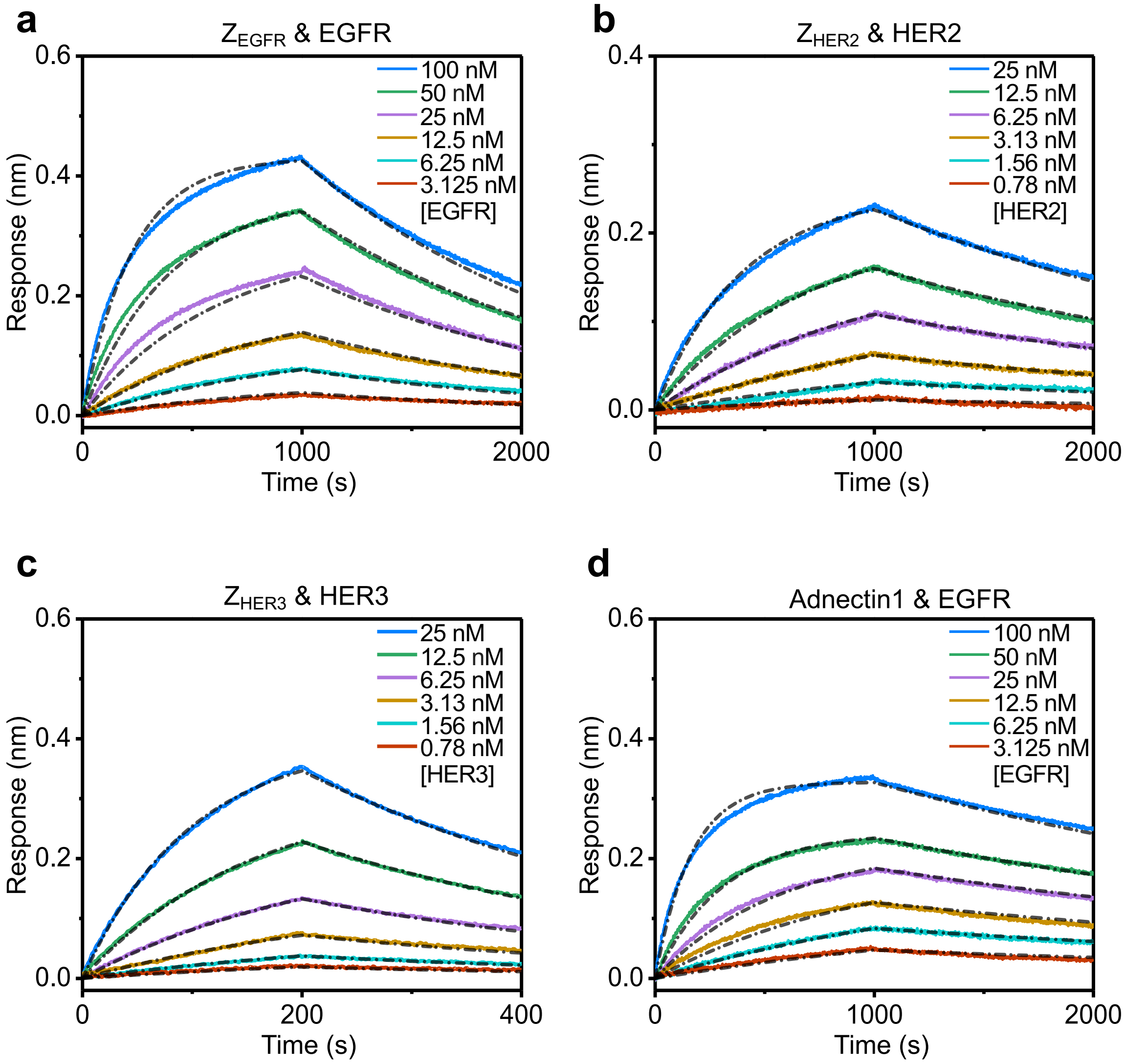

<u>**Supplementary Figure S7.**</u> **Fits of label-free BLI experiments of the binder-ligand receptor when the binder was immobilized onto the BLI sensor surface**. The FortéBio Octet Data Analysis software (FortéBio) was used to fit binding curves. The dotted black line represents the fitting curve.

<u>Supplementary Table S11.</u> BLI-determined kinetic rate constants of association (k_{on} and k_{off}), and equilibrium dissociation constants (K_D) of the binder-ligand interactions with the binders attached to the BLI chip surface and the ligands added to the wells. The running buffer consisted of 20 mM Tris-HCl, 300 mM KCl, and 1 mg/ml bovine serum albumin (BSA), pH 7.5. Values of all constants, which were derived using global fits **(Experimental Section)**, indicate mean ± s.e.m. The average values indicate the mean ± s.d., which were acquired using n = 3 independent BLI experiments.

	$k_{on} \times 10^{-4}$ ($M^{-1}s^{-1}$)		$k_{off} \times 10^{4}$ (s^{-1})		K_D (nM)	
	**Single cycle**	**Average**	**Single cycle**	**Average**	**Single cycle**	**Average**
Z_{EGFR} & EGFR	5.21 ± 0.02<br>6.35 ± 0.03<br>4.92 ± 0.02	5.49 ± 0.76	2.48 ± 0.01<br>3.87 ± 0.02<br>3.53 ± 0.02	3.29 ± 0.72	4.76 ± 0.03<br>6.10 ± 0.04<br>7.17 ± 0.04	6.0 ± 1.2
Z_{HER2} & HER2	6.09 ± 0.04<br>6.24 ± 0.03<br>8.28 ± 0.03	6.87 ± 1.23	3.09 ± 0.05<br>3.48 ± 0.02<br>4.45 ± 0.01	3.68 ± 0.70	5.07 ± 0.05<br>5.58 ± 0.04<br>5.38 ± 0.02	5.4 ± 0.3
Z_{HER3} & HER3	22.09 ± 0.09<br>22.24 ± 0.09<br>29.82 ± 0.12	24.72 ± 4.42	18.82 ± 0.03<br>18.11 ± 0.03<br>26.59 ± 0.09	21.17 ± 4.70	8.52 ± 0.04<br>8.41 ± 0.04<br>8.92 ± 0.05	8.5 ± 0.4
Adnectin1 & EGFR	6.24 ± 0.02<br>5.80 ± 0.02<br>5.78 ± 0.02	5.94 ± 0.26	3.55 ± 0.01<br>3.59 ± 0.01<br>3.03 ± 0.01	3.39 ± 0.31	5.68 ± 0.03<br>6.18 ± 0.03<br>5.24 ± 0.02	5.7 ± 0.5

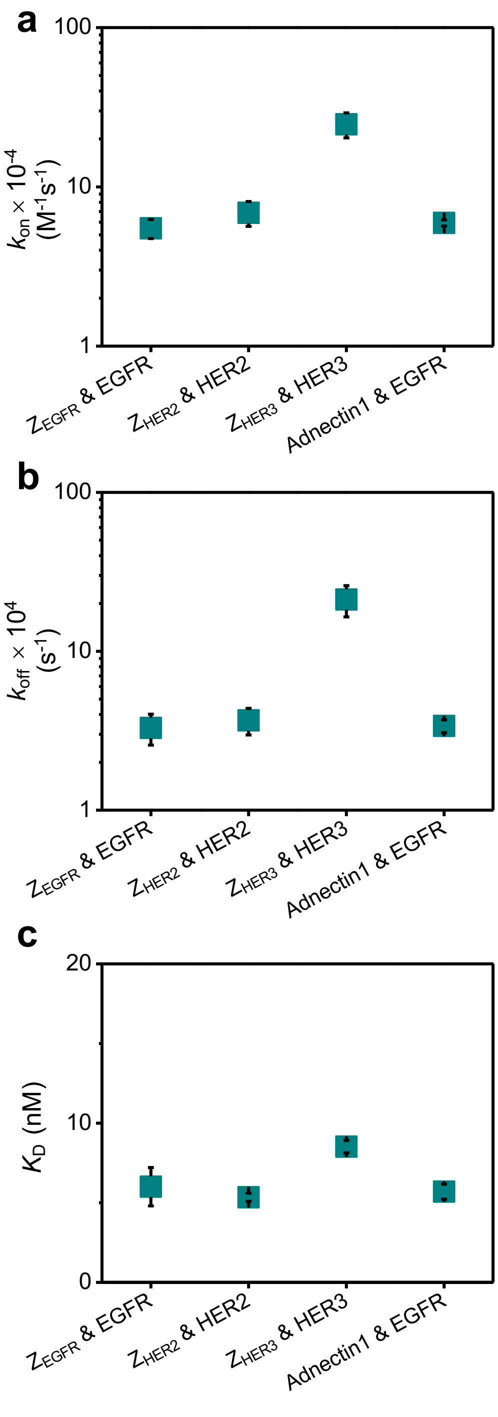

Supplementary Figure S8. The kinetic rate constants of association and dissociation, and the equilibrium dissociation constants of binder-ligand interactions, were derived from real-time BLI measurements. These experiments involved interactions between binders immobilized onto the BLI sensor surface and their cognate proteins. **(a)** The kinetic rate constants of association, k_{on}. **(b)** The kinetic rate constants of dissociation, k_{off}. **(c)** The equilibrium dissociation constants, K_D. Data points represent mean ± s.d. obtained from n = 3 distinct experiments. They can be found in **Supplementary Table S8**.

8. Equilibrium dissociation constants (K_D) of binder-protein ligand interactions reported by other research groups under different experimental circumstances.

Supplementary Table S12. Affibody Molecules Target EGFR Family Reported by other research teams.

Target	Affibody	Affinity	References
EGFR	$Z_{EGFR:1907}$	5.4 nM (SPR)	13
HER2	$Z_{HER2:342}$	22 pM (SPR)	14
HER3	$Z_{HER3:05413}$	0.78 nM (SPR)	15

[13]In this reference, the ectodomain of EGFR (EGFR-ECD), which was diluted in 10 mM NaAc, pH 4.5, was immobilized onto the SPR sensor surface. The running buffer consisted of 10 mM HEPES, 150 mM NaCl, 3.4 mM ethylenediaminetetraacetic acid (EDTA), and 0.005% surfactant P20, at pH 7.4.

[14]In this reference, the ectodomain of HER2 (HER2-ECD) was immobilized onto the SPR sensor surface. The running buffer consisted of 137 mM NaCl, 2.7 mM KCl, 8 mM Na_2HPO_4, and 2 mM KH_2PO_4, at pH 7.4.

[15] In this reference, the ectodomain of HER3 (HER3-ECD) fused to the human IgG Fc-region (HER3-ECD-Fc) was immobilized onto the SPR sensor surface. The running buffer included 137 mM NaCl, 2.7 mM KCl, 8 mM Na_2HPO_4, 2 mM KH_2PO_4, and 0.1% Pluronic F108 NF surfactant, pH 7.4.

Supplementary Table S13. Monobody Molecules Affinity Reported in the Literature

Target	Monobody	Affinity	References
EGFR	Adnectin 1	2 nM	3

[3] In this reference, the EGFR ectodomain (EGFR-ECD) was fused with the human IgG Fc region (EGFR-ECD-Fc) and immobilized onto the SPR sensor surface. The running buffer included 10 mM HEPES, 150 mM NaCl, and 0.05% Surfactant P20, pH 7.4.

Supplementary Table S14. **Molecules Target EGFR Reported in the Literature**

Target	Protein	Affinity	References
EGFR	EGF	7.6 ± 1.2 nM (SPR)	16
	TGF-α	9.6 ± 1.3 nM (SPR)	16

[16] In this reference, the ectodomain of EGFR (EGFR-ECD) was immobilized onto the surface of the SPR sensor. The running buffer consisted of 137 mM NaCl, 2.7 mM KCl, 8 mM Na_2HPO_4, and 2 mM KH_2PO_4, at pH 7.4.

9. BLI tests with the small proteins immobilized onto the sensor surface show no response

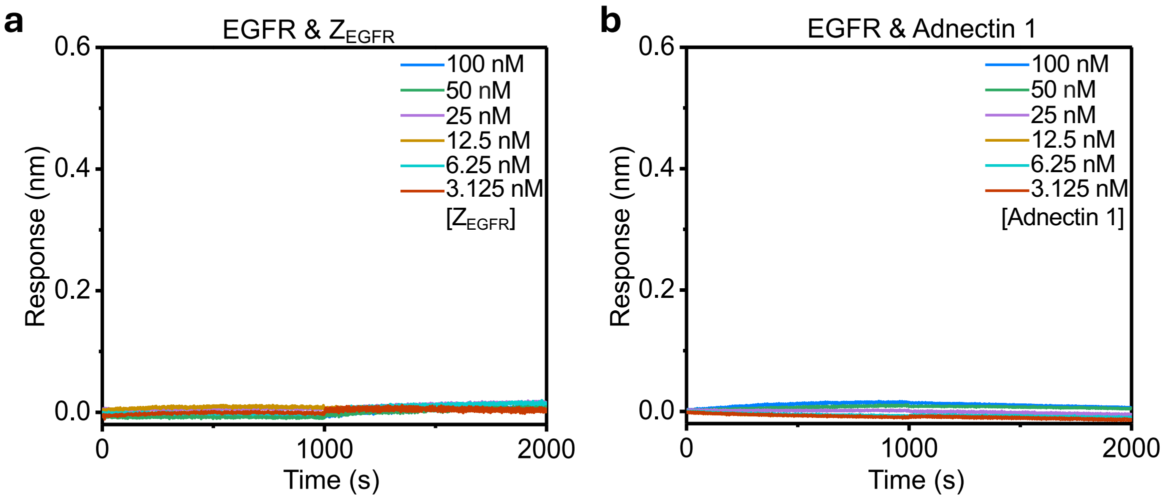

Supplementary Figure S9. **Real-time, label-free BLI experiments of the ligand-binder interaction when the protein ligand was immobilized onto the BLI sensor surface. (a)** EGFR-Z_{EGFR}. **(b)** EGFR-Adnectin1. In both cases, EGFR was attached to the sensor surface. The binder was either Z_{EGFR} (a) or Adnectin1 (b). BLI sensorgrams cannot show the association and dissociation phases. For each panel, 50 nM biotinylated ligand was loaded on streptavidin-coated BLI sensors for 5 min and dipped into the running buffer containing one of the six twofold serial dilution concentrations of the binder for the association phase. Sensors were then transferred to a protein binder-free running buffer for the dissociation phase.

10. Surface plasmon resonance (SPR) experiments as confirmatory tests of the BLI measurements.

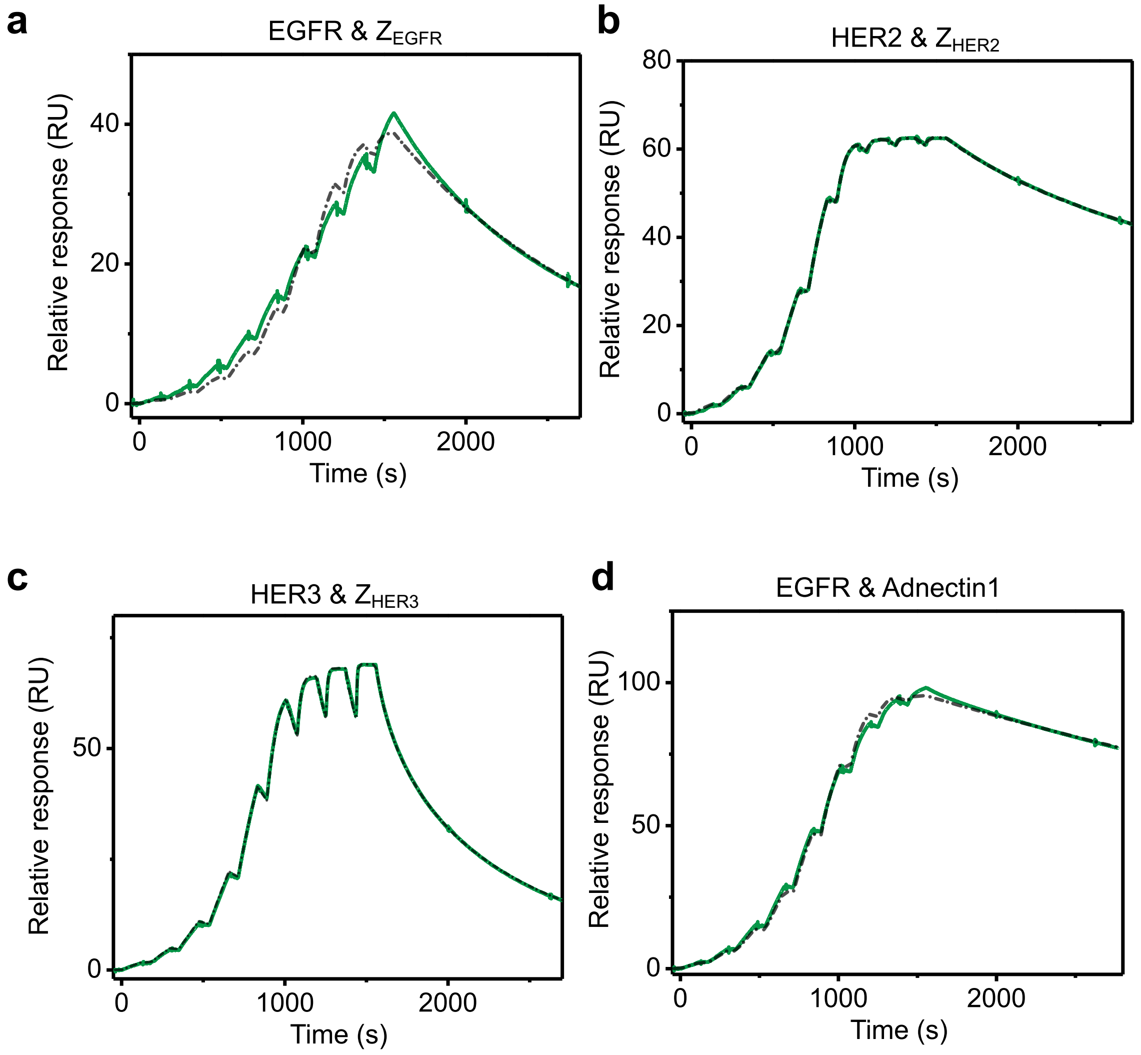

Supplementary Figure S10. Representative single-cycle label-free SPR runs and their corresponding fits of the ligand–binder interactions with the binders added to the well and the ligands attached to the SPR chip surface. A single-cycle kinetic assay was performed due to the analyte's incomplete dissociation. The ligand was immobilized onto Cytiva Series CM5 chips. The association and dissociation phases were probed in real time. A nine-point, two-fold serial dilution of the binder was injected in single-cycle kinetics, with 120 s association phases and short dissociation times between injections. **(a)** EGFR was immobilized on the chip surface, and nine two-fold dilutions of Z_{EGFR} (1.56-400 nM) were sequentially injected. **(b)** HER2 was immobilized on the chip surface, and nine two-fold dilutions of Z_{HER2} (0.20-50 nM) were sequentially injected. **(c)** HER3 was immobilized on the chip surface, and nine two-fold dilutions of Z_{HER3} (0.20-50 nM) were sequentially injected. **(d)** EGFR was immobilized on the chip surface, and nine two-fold dilutions of Adnectin1 (1.56-400 nM) were sequentially injected. The SPR runs are illustrated by green lines. The fits are shown in black dotted lines.

Supplementary Table S15. SPR-determined kinetic rate constants of association (k_{on} and k_{off}), and equilibrium dissociation constants (K_D) of the binder-ligand interactions with the binders added to the well and the ligands attached to the SPR chip surface. The running buffer consisted of 20 mM Tris-HCl, 300 mM KCl, 1 mg/ml bovine serum albumin (BSA), and 0.05% (v/v) Tween 20, at pH 7.5. The single-cycle values of the kinetic rate constants indicate mean ± s.e.m. using individual cycle fits. The average values are reported as the mean ± standard deviation (s.d.), derived from *n* = 3 independent SPR cycles.

	$k_{on} \times 10^{-4}$ ($M^{-1}s^{-1}$)		$k_{off} \times 10^{4}$ (s^{-1})		K_D (nM)	
	**Single cycle**	**Average**	**Single cycle**	**Average**	**Single cycle**	**Average**
Z_{EGFR} & EGFR	14.01 ± 0.03<br>13.77 ± 0.03<br>13.72 ± 0.03	13.83 ± 0.16	7.35 ± 0.01<br>7.35 ± 0.01<br>7.29 ± 0.01	7.33 ± 0.03	5.24<br>5.34<br>5.31	5.30 ± 0.05
Z_{HER2} & HER2	465.0 ± 0.4<br>476.2 ± 0.4<br>481.2 ± 0.5	473.8 ± 8.9	4.54 ± 0.01<br>4.55 ± 0.01<br>4.50 ± 0.01	4.53 ± 0.02	0.98×10^{-1}<br>0.96×10^{-1}<br>0.94×10^{-1}	$(0.96 \pm 0.02) \times 10^{-1}$
Z_{HER3} & HER3	647.4 ± 0.1<br>654.5 ± 0.7<br>627.5 ± 1.0	642.8 ± 14.5	43.6 ± 0.01<br>44.7 ± 0.05<br>45.9 ± 0.07	44.76 ± 1.15	0.67<br>0.68<br>0.73	0.70 ± 0.03
Adnectin1 & EGFR	13.3 ± 0.01<br>13.1 ± 0.01<br>12.2 ± 0.01	12.85 ± 0.60	1.65 ± 0.01<br>1.64 ± 0.01<br>1.73 ± 0.01	1.67 ± 0.05	1.25<br>1.25<br>1.42	1.30 ± 0.10

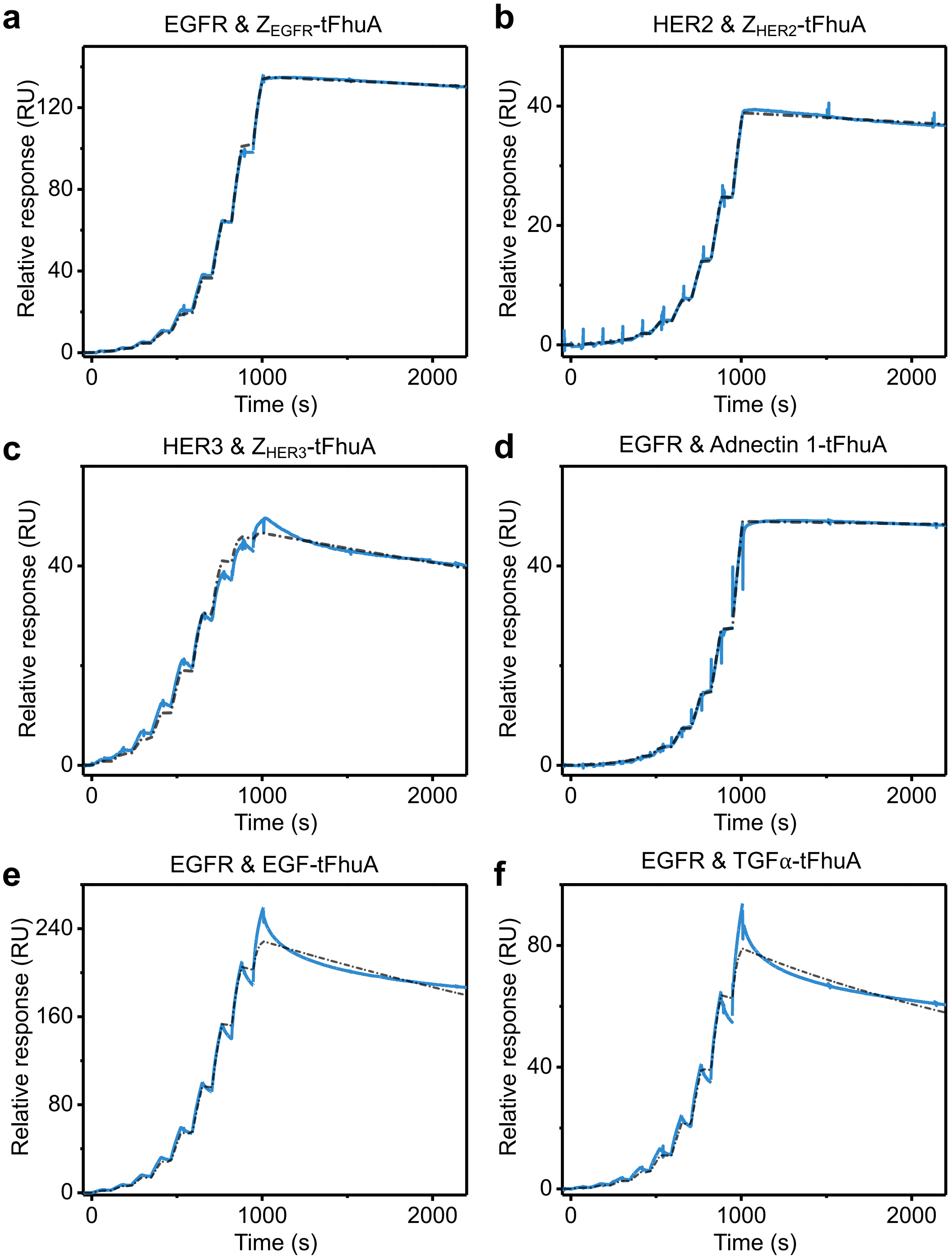

<u>Supplementary Figure S11.</u> Experimental curves and their fits of label-free SPR experiments of the ligand – membrane protein when the ligand was immobilized onto the SPR chip surface. A single-cycle kinetic assay was performed due to the analyte's incomplete dissociation. The ligand was immobilized onto Cytiva Series streptavidin chips. The association and dissociation phases were probed in real time. A nine-point, two-fold serial dilution of the membrane protein was injected in single-cycle kinetics, with 60 s association phases and short dissociation times between injections. **(a)** EGFR was immobilized on the chip surface, and nine two-fold dilutions of Z_{EGFR}-tFhuA (3.9-1000 nM) were sequentially injected. **(b)** HER2 was

immobilized on the chip surface, and nine two-fold dilutions of Z_{HER2}-tFhuA (0.97-250 nM) were sequentially injected. **(c)** HER3 was immobilized on the chip surface, and nine two-fold dilutions of Z_{HER3}-tFhuA (0.97-250 nM) were sequentially injected. **(d)** EGFR was immobilized on the chip surface, and nine two-fold dilutions of Adnectin1-tFhuA (3.9-1000 nM) were sequentially injected. **(e)** EGFR was immobilized on the chip surface, and nine two-fold dilutions of EGF-tFhuA (7.81-2000 nM) were sequentially injected. **(f)** EGFR was immobilized on the chip surface, and nine two-fold dilutions of TGFα-tFhuA (7.81-2000 nM) were sequentially injected. The fits are shown in black dotted line.

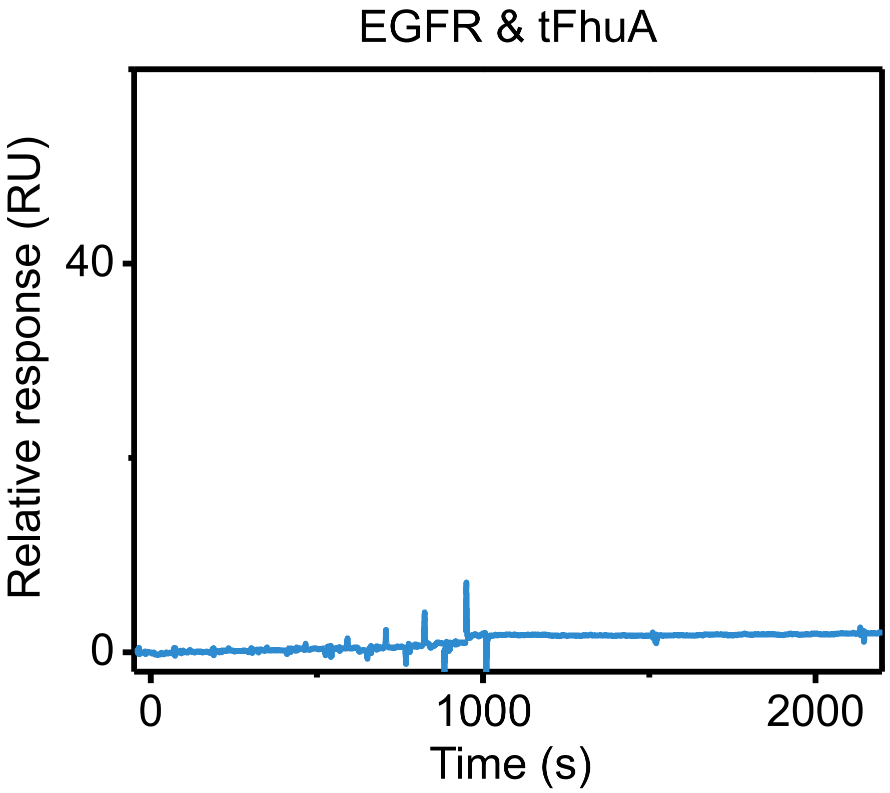

Supplementary Figure S12. Negative control experiment where EGFR was immobilized onto the SPR chip surface and tFhuA-containing proteomicells were injected using the microfluidic systems of the instrument. Nine two-fold dilutions of tFhuA (3.9-1000 nM) were sequentially injected.

<u>Supplementary Table S16.</u> SPR-determined kinetic rate constants of association (k_{on}) and dissociation (k_{off}), and equilibrium dissociation constants (K_D) for the binding of ligand – membrane protein interactions using the ligand attached to the SPR chip surface. The running buffer consisted of 20 mM Tris-HCl, 300 mM KCl, 1 mg/ml bovine serum albumin (BSA), 0.05% (v/v) Tween 20, and 0.2% (w/v) DDM, at pH 7.5. The single-cycle values indicate mean ± s.e.m. The average values indicate mean ± s.d. using n = 3 independent SPR experiments.

	$k_{on} \times 10^{-4}$ ($M^{-1}s^{-1}$)		$k_{off} \times 10^{4}$ (s^{-1})		K_D (nM)	
	**Single cycle**	**Average**	**Single cycle**	**Average**	**Single cycle**	**Average**
EGFR & ZEGFR-tFhuA	1.89 ± 0.01<br>1.60 ± 0.01<br>1.86 ± 0.01	1.78 ± 0.02	0.29 ± 0.01<br>0.33 ± 0.01<br>0.37 ± 0.01	0.33 ± 0.04	1.54<br>2.05<br>2.01	1.87 ± 0.28
HER2 & ZHER2-tFhuA	3.80 ± 0.01<br>3.82 ± 0.01<br>3.35 ± 0.01	3.65 ± 0.27	0.42 ± 0.01<br>0.45 ± 0.01<br>0.44 ± 0.01	0.44 ± 0.01	1.11<br>1.17<br>1.32	1.20 ± 0.11
HER3 & ZHER3-tFhuA	22.52 ± 0.04<br>31.27 ± 0.05<br>29.41 ± 0.04	30.06 ± 1.04	1.36 ± 0.01<br>1.15 ± 0.01<br>1.46 ± 0.01	1.32 ± 0.16	0.46<br>0.37<br>0.50	0.44 ± 0.07
EGFR & Adnectin1-tFhuA	0.41 ± 0.01<br>0.55 ± 0.01<br>0.60 ± 0.01	0.52 ± 0.10	0.10 ± 0.01<br>0.12 ± 0.01<br>0.37 ± 0.01	0.20 ± 0.15	2.46<br>2.10<br>6.15	3.57 ± 2.24
EGFR & EGF-tFhuA	2.06 ± 0.01<br>1.88 ± 0.01<br>1.99 ± 0.01	1.98 ± 0.09	2.67 ± 0.01<br>2.03 ± 0.01<br>1.83 ± 0.01	2.18 ± 0.44	13.00<br>10.75<br>9.23	10.99 ± 1.90
EGFR & TGFa-tFhuA	2.06 ± 0.03<br>1.21 ± 0.01<br>1.19 ± 0.01	1.49 ± 0.50	3.07 ± 0.02<br>2.13 ± 0.01<br>3.49 ± 0.01	2.90 ± 0.70	14.89<br>17.63<br>29.42	20.65 ± 7.72

11. Supplementary references

(1) Locher, K. P.; Rees, B.; Koebnik, R.; Mitschler, A.; Moulinier, L.; Rosenbusch, J. P.; Moras, D. Transmembrane Signaling across the Ligand-Gated FhuA Receptor: Crystal Structures of Free and Ferrichrome-Bound States Reveal Allosteric Changes. *Cell* **1998**, *95* (6), 771-778. DOI: 10.1016/S0092-8674(00)81700-6 (accessed 2024/12/22).

(2) Eigenbrot, C.; Ultsch, M.; Dubnovitsky, A.; Abrahmsén, L.; Härd, T. Structural basis for high-affinity HER2 receptor binding by an engineered protein. *Proceedings of the National Academy of Sciences* **2010**, *107* (34), 15039-15044. DOI: doi:10.1073/pnas.1005025107.

(3) Ramamurthy, V.; Krystek, Stanley R.; Bush, A.; Wei, A.; Emanuel, Stuart L.; Das Gupta, R.; Janjua, A.; Cheng, L.; Murdock, M.; Abramczyk, B.; et al. Structures of Adnectin/Protein Complexes Reveal an Expanded Binding Footprint. *Structure* **2012**, *20* (2), 259-269. DOI: https://doi.org/10.1016/j.str.2011.11.016.

(4) Ferguson, K. M.; Berger, M. B.; Mendrola, J. M.; Cho, H. S.; Leahy, D. J.; Lemmon, M. A. EGF activates its receptor by removing interactions that autoinhibit ectodomain dimerization. *Mol. Cell* **2003**, *11* (2), 507-517. DOI: 10.1016/s1097-2765(03)00047-9 From NLM.

(5) Huang, Y.; Ognjenovic, J.; Karandur, D.; Miller, K.; Merk, A.; Subramaniam, S.; Kuriyan, J. A molecular mechanism for the generation of ligand-dependent differential outputs by the epidermal growth factor receptor. *eLife* **2021**, *10*, e73218. DOI: 10.7554/eLife.73218.

(6) Kyte, J.; Doolittle, R. F. A simple method for displaying the hydropathic character of a protein. *J. Mol. Biol.* **1982**, *157* (1), 105-132.

(7) Wolfe, A. J.; Mohammad, M. M.; Cheley, S.; Bayley, H.; Movileanu, L. Catalyzing the Translocation of Polypeptides through Attractive Interactions. *J. Am. Chem. Soc.* **2007**, *129* (45), 14034-14041.

(8) Mayse, L. A.; Imran, A.; Larimi, M. G.; Cosgrove, M. S.; Wolfe, A. J.; Movileanu, L. Disentangling the recognition complexity of a protein hub using a nanopore. *Nature Commun.* **2022**, *13* (1), 978. DOI: 10.1038/s41467-022-28465-8.

(9) Ahmad, M.; Ha, J. H.; Mayse, L. A.; Presti, M. F.; Wolfe, A. J.; Moody, K. J.; Loh, S. N.; Movileanu, L. A generalizable nanopore sensor for highly specific protein detection at single-molecule precision. *Nature Commun.* **2023**, *14* (1), 1374. DOI: 10.1038/s41467-023-36944-9 From NLM.

(10) Conlan, S.; Bayley, H. Folding of a monomeric porin, OmpG, in detergent solution. *Biochemistry* **2003**, *42* (31), 9453-9465.

(11) Fasman, G. D. *Circular Dichroism and the Conformational Analysis of Biomolecules*; Springer 1996. DOI: https://doi.org/10.1007/978-1-4757-2508-7.

(12) Bonhivers, M.; Desmadril, M.; Moeck, G. S.; Boulanger, P.; Colomer-Pallas, A.; Letellier, L. Stability studies of FhuA, a two-domain outer membrane protein from Escherichia coli. *Biochemistry* **2001**, *40* (8), 2606-2613.

(13) Friedman, M.; Orlova, A.; Johansson, E.; Eriksson, T. L. J.; Höidén-Guthenberg, I.; Tolmachev, V.; Nilsson, F. Y.; Ståhl, S. Directed Evolution to Low Nanomolar Affinity of a Tumor-Targeting Epidermal Growth Factor Receptor-Binding Affibody Molecule. *Journal of Molecular Biology* **2008**, *376* (5), 1388-1402. DOI: https://doi.org/10.1016/j.jmb.2007.12.060.

(14) Orlova, A.; Magnusson, M.; Eriksson, T. L. J.; Nilsson, M.; Larsson, B.; Höidén-Guthenberg, I.; Widström, C.; Carlsson, J. r.; Tolmachev, V.; Ståhl, S.; Nilsson, F. Y. Tumor Imaging Using a Picomolar Affinity HER2 Binding Affibody Molecule. *Cancer Research* **2006**, *66* (8), 4339-4348. DOI: 10.1158/0008-5472.Can-05-3521 (accessed 2/17/2025).

(15) Kronqvist, N.; Malm, M.; Göstring, L.; Gunneriusson, E.; Nilsson, M.; Höidén Guthenberg, I.; Gedda, L.; Frejd, F. Y.; Ståhl, S.; Löfblom, J. Combining phage and staphylococcal surface display for generation of ErbB3-specific Affibody molecules. *Protein Engineering, Design and Selection* **2010**, *24* (4), 385-396. DOI: 10.1093/protein/gzq118 (accessed 2/17/2025).

(16) Wang, Y.; Zhang, C.; Zhang, Y.; Fang, H.; Min, C.; Zhu, S.; Yuan, X. C. Investigation of phase SPR biosensor for efficient targeted drug screening with high sensitivity and stability. *Sensors and Actuators B: Chemical* **2015**, *209*, 313-322. DOI: https://doi.org/10.1016/j.snb.2014.11.134.
